# Replication and Recombination Factors Contributing to Recombination-Dependent Bypass of DNA Lesions by Template Switch

**DOI:** 10.1371/journal.pgen.1001205

**Published:** 2010-11-11

**Authors:** Fabio Vanoli, Marco Fumasoni, Barnabas Szakal, Laurent Maloisel, Dana Branzei

**Affiliations:** 1 Fondazione IFOM, Istituto FIRC di Oncologia Molecolare, Milan, Italy; 2 Università degli Studi di Milano, Milan, Italy; 3 CEA, DSV, iRCM, SIGRR, LRGM, and CNRS, UMR 217, Fontenay-aux-Roses, France; Brandeis University, United States of America

## Abstract

Damage tolerance mechanisms mediating damage-bypass and gap-filling are crucial for genome integrity. A major damage tolerance pathway involves recombination and is referred to as template switch. Template switch intermediates were visualized by 2D gel electrophoresis in the proximity of replication forks as X-shaped structures involving sister chromatid junctions. The homologous recombination factor Rad51 is required for the formation/stabilization of these intermediates, but its mode of action remains to be investigated. By using a combination of genetic and physical approaches, we show that the homologous recombination factors Rad55 and Rad57, but not Rad59, are required for the formation of template switch intermediates. The replication-proficient but recombination-defective *rfa1-t11* mutant is normal in triggering a checkpoint response following DNA damage but is impaired in X-structure formation. The Exo1 nuclease also has stimulatory roles in this process. The checkpoint kinase, Rad53, is required for X-molecule formation and phosphorylates Rad55 robustly in response to DNA damage. Although Rad55 phosphorylation is thought to activate recombinational repair under conditions of genotoxic stress, we find that Rad55 phosphomutants do not affect the efficiency of X-molecule formation. We also examined the DNA polymerase implicated in the DNA synthesis step of template switch. Deficiencies in translesion synthesis polymerases do not affect X-molecule formation, whereas DNA polymerase δ, required also for bulk DNA synthesis, plays an important role. Our data indicate that a subset of homologous recombination factors, together with DNA polymerase δ, promote the formation of template switch intermediates that are then preferentially dissolved by the action of the Sgs1 helicase in association with the Top3 topoisomerase rather than resolved by Holliday Junction nucleases. Our results allow us to propose the choreography through which different players contribute to template switch in response to DNA damage and to distinguish this process from other recombination-mediated processes promoting DNA repair.

## Author Summary

Completion of DNA replication is essential for cellular survival. Both endogenous processes and exogenous DNA damage can lead to lesions that impede DNA replication or result in an accumulation of DNA gaps. Recombination plays an important role in facilitating replication completion under conditions of replication stress or DNA damage. One DNA damage tolerance mechanism involving recombination factors, template switch, uses the information on the newly synthesized sister chromatid to fill in the gaps arising during replication under damaging conditions. This process leads to the formation of repair structures involving sister chromatid junctions in the proximity of replication forks. The template switch structures can be detected by 2D gel electrophoresis of replication intermediates as cruciform, X-shaped intermediates. Additional factors and regulatory pathways are required for the resolution of such structures to prevent their toxic effects. In this work, we have dissected the recombination/replication factors required for the formation of template switch intermediates. Another recombination mechanism, which has been implicated in the restart of collapsed forks, is break-induced replication (BIR). This study allows us to identify the core factors required for template switch and to distinguish this process from other recombination-mediated processes promoting DNA repair.

## Introduction

Proliferating cells are constantly exposed to DNA damage from both endogenous and exogenous sources. These DNA lesions can cause replication fork collapse and cell cycle arrest thereby posing a serious threat to genome integrity. To avoid the catastrophic consequences associated with fork demise, cells have evolved multiple mechanisms by which arrested or stalled replication forks can be rescued. These mechanisms are collectively referred to as DNA damage tolerance (DDT) mechanisms and involve factors belonging to two main repair pathways: the *RAD52* homologous recombination (HR) and the *RAD6/RAD18* post-replication repair (PRR) pathways [1], [2]. The DDT mechanisms available in a cell are largely divided into two classes. One utilizes a combination of replicative and translesion synthesis (TLS) polymerases to replicate across the lesion, and in such situations the bypass can occur either in error-free or in error-prone manners [3], [4]. The other DDT mechanism copies the information from undamaged segments of the genome, usually in an error-free manner and is referred to as template switch [2], [5]–[7].

The mechanism, mode of action and factors implicated in template switch remain largely unknown [2]. Since template switch refers to a damage bypass process that operates in an error-free manner, it had been presumed to resemble and/or to involve recombination. Accordingly, distinct mechanisms involving recombination were proposed to account for template switch. One replication restart model of template switch, known also as the chicken foot model, proposes that the damage-bypass occurs at the site of fork stalling and involves pairing of the newly synthesized sister chromatids and replication fork regression [5], [8], [9]. The other model also proposes pairing of the newly synthesized sister chromatids at the fork or behind the fork in a manner that resembles the strand-exchange model of HR and leads to formation of sister chromatid junctions (SCJs) [6], [7], [10]. Whether template switch operates primarily at the fork or behind the fork could significantly affect the intermediate template switch DNA structure and has been an issue of debate [2], [3]. Recent findings showing that restriction of the *RAD18* pathway to G2 still supports lesion tolerance [11], and that, during replication under damaging conditions when DDT factors are limiting, gaps accumulate behind the replication forks [12], strongly corroborate the idea that template switch operates mainly in the rear of replication forks. Together with these findings, genetic and physical evidence have provided support for the model by which template switch occurs via recombination-like intermediates involving sister chromatid junctions (SCJs) [6], [10], [13].

In the recombination-like mode of template switch, annealing between the two newly synthesized sister-chromatids is expected to give rise to a D-loop recombination intermediate, which upon extension will lead to transient, hemicatenane-like or pseudo-double Holliday Junctions (HJs) structures (Figure S1) or to double HJs [14], [15]. In budding yeast, X-shaped intermediates with the expected biochemical properties of pseudo-double HJs and not of reversed forks or canonical HJs have been visualized during replication of damaged templates by using the 2D gel electrophoresis technique [10]. The resolution/dissolution of these DNA X-structures requires primarily the activity of the RecQ helicase Sgs1 (BLM in mammalian cells) and of the topoisomerase Top3 rather than that of Holliday junction nucleases [10], [16]–[18].

If template switch operates mainly behind the forks to promote gap-filling, then factors required to promote replication completion and filling of gaps, such as those induced by UV irradiation, are expected to be required as well for the formation of template switch intermediates. Previous work in *S. cerevisiae* has shown that, following UV irradiation, DNA is initially synthesized as small discontinuous fragments, which are later converted to higher molecular-weight pieces similar in size to DNA from unirradiated cells [19], [20]. Subsequent work has shown that these UV-induced gaps can be filled in a manner dependent on HR factors as well as proteins such as Rad18, Rad5 and Mms2, implicated in the error-free class of the PRR pathway [21]–[23]. Notably, both HR and error-free PRR factors have been shown to contribute to the formation of these template switch damage-bypass intermediates involving SCJs [10], [13], [15], [24]. Altogether, these findings suggest that template switch represents a specific class of recombination process, involving in addition to traditional HR factors, other sets of enzymes with affinity for single-stranded (ss) DNA such as Rad5 and Rad18 [25], [26]. The visualization of these intermediates in the proximity of replication forks, together with the evidence that these events are likely to be post-replicative, operating on the gaps left behind the forks [11]–[13], suggest that template switch takes place during chromosomal replication although it does not interfere with the DNA synthesis process occurring at the replication fork.

Thus, in terms of genetic requirements for error-free PRR and HR factors, post-replicative gap-repair and template switch appear to be similar. However, the exact role of HR and PRR factors in the formation/stabilization of template switch intermediates, the other players involved in this process and how these factors are coordinated with one another as well as with other gap-processing activities remain largely unknown. In this study, we planned to address these questions by dissecting the role of different factors in the formation of template switch intermediates. We analyzed factors that distinctly affect HR (Rad55, Rad57, Rfa1, Rad59), factors implicated in gap processing and in the DNA damage/checkpoint response (Exo1, Rfa1) as well as the contribution of different DNA polymerase activities to the DNA synthesis step of template switch.

HR mechanisms have been primarily modeled to explain double strand break (DSB) repair, and it has been demonstrated that the ends of a DSB are resected to expose 3′-single stranded (ss) tails that are bound by Rad51 and invade homologous duplex DNA, leading to a D-loop structure that can be subsequently extended and serve as a primer for DNA synthesis [27]–[29]. In *S. cerevisiae*, Rad52 plays an essential role in mediating strand exchange: the ssDNA is normally coated by the ssDNA binding protein RPA; Rad52, which interacts with both Rad51 and RPA, overcomes the inhibitory role of RPA, recruits Rad51, and promotes the formation of active Rad51 nucleofilaments that catalyze strand invasion [30]. The Rad51 paralogues, Rad55 and Rad57, form a heterodimeric complex that interacts with Rad51 and has ssDNA binding activity but apparently no recombinase activity [31]. Similar to Rad52, Rad55-Rad57 acts substoichiometrically to Rad51 to overcome the inhibitory role of RPA on Rad51-mediated strand exchange, indicative of a recombination mediator activity, although the mechanism of mediation is unknown [30], [31]. Genetic and biochemical data suggests that Rad55-Rad57 also acts to stabilize the assembled Rad51 nucleofilaments [32]. The recombination defects of *rad55*, *rad57* are not always similar to the ones of *rad51*. Notably, in spite of the generally much weaker phenotypes of *rad55*, *rad57* mutants in HR as compared to *rad51*, *rad57* cells are much more defective in spontaneous sister chromatid recombination (SCR) than *rad51* [33]. Furthermore, in contrast to the defects of *rad55* and *rad57* mutants in DSB repair, which are suppressed by *RAD51* overexpression, their SCR defect is only partly suppressed, suggesting that Rad55-Rad57 roles in DSB repair are distinct from their role in spontaneous SCR, which likely initiates from ssDNA gaps formed during replication [33]. Studies of the mammalian Rad51 paralogs Rad51C and Xrcc3 and of the *rad57* mutants of *Schizosaccharomyces pombe* also suggested a possible role for Rad55-Rad57 in late recombination events, for instance by promoting the resolution of recombination intermediates or the displacement of the invading strand [34]–[37]. In budding yeast, the Rad55 protein is phosphorylated in a checkpoint-dependent manner under conditions of DNA damage, and this modification appears important for Rad55 function upon genome-wide genotoxic stress [38]. However, the effect of Rad55 phosphorylation on recombination and the recombination-mediator function of Rad55-Rad57 remain to be seen. The budding yeast Rad59 protein has similarity to the N-terminal region of Rad52 and is implicated in a subset of HR events, including spontaneous and damage-induced sister chromatid exchanges [39]–[42] and certain pathways of break-induced replication (BIR) [43], [44]—an efficient HR process required to initiate replication when only one end of a DSB shares homology with a template [45]–[49]. In vitro studies have shown that Rad59 promotes strand annealing but is unable to stimulate Rad51-mediated strand exchange [50]. Understanding the contribution of different HR proteins to template switch will likely help elucidate the precise mechanism of this process and provide insights into how stalling or collapse of the replication fork triggers different recombination-mediated mechanisms in order to promote replication completion.

Cells have a number of replicative and specialized TLS polymerases that participate in DNA replication as well as in different DNA repair events, but the replication activities required to promote the DNA synthesis step of template switch are presently unknown. The DNA polymerases α, δ, and ε (Polα, Polδ, and Polε) are the major replicative polymerases in eukaryotic cells, required to replicate DNA with high speed and fidelity [51]. Polα is tightly associated with the primase and is required for initiation of DNA synthesis on the leading strand as well as for the continuous synthesis of Okazaki fragments on the lagging strand. Although studies of Simian Virus (SV40) DNA replication showed Polδ to be required for the extension of both leading and lagging strands [51], and the polymerase activity of Polε in yeast cells is not essential for cell viability [52], [53], it is now generally agreed that both Polδ and Polε contribute to cellular DNA replication. Furthermore, mutational analyses of yeast suggest a differential involvement of Polδ and Polε in the synthesis of lagging and leading strands, respectively [54], [55]. Loading of Polδ requires the proliferating cell nuclear antigen (PCNA) and replication factor C (RFC), which function as a sliding clamp and a clamp loader, respectively. In addition, PCNA is also required for processive DNA synthesis by Polδ [51] and stimulates both Polε and Polδ in vitro [56]. In contrast to the replicative DNA polymerases, TLS polymerases such as Polη, Polζ and Rev1 in budding yeast, as well as their mammalian counterparts, have more open active sites, a property that allows these enzymes to accommodate bulky lesions and to promote replication through damaged templates [1], [4]. It has been proposed that Polη has an additional role in promoting DNA synthesis during HR-mediated repair of DSBs [57], [58].

In the present work we have examined the role of different recombination and replication factors in the formation of template switch intermediates during replication of damaged templates in vivo. By using a combination of genetic and physical assays, we show that factors implicated in the strand invasion step of HR, but not the strand annealing factor Rad59, which is not essential for the strand exchange reaction, are required for the formation of the X-shaped template switch intermediates involving SCJs. Other factors, such as Exo1, which is known to affect processing of recombination and replication intermediates, also play a role in promoting template switch. We demonstrate that TLS polymerases do not affect the efficiency of this process, while Polδ plays a major role in the DNA synthesis step of template switch. We thus identify a dual role for Polδ in genome replication and replication-associated repair and discuss mechanisms through which this functional versatility may be achieved.

## Results

### Physical assay to analyze the genetic requirements for template switch–mediated damage-bypass of chromosomal lesions in *S. cerevisiae*

**Figure 1:**
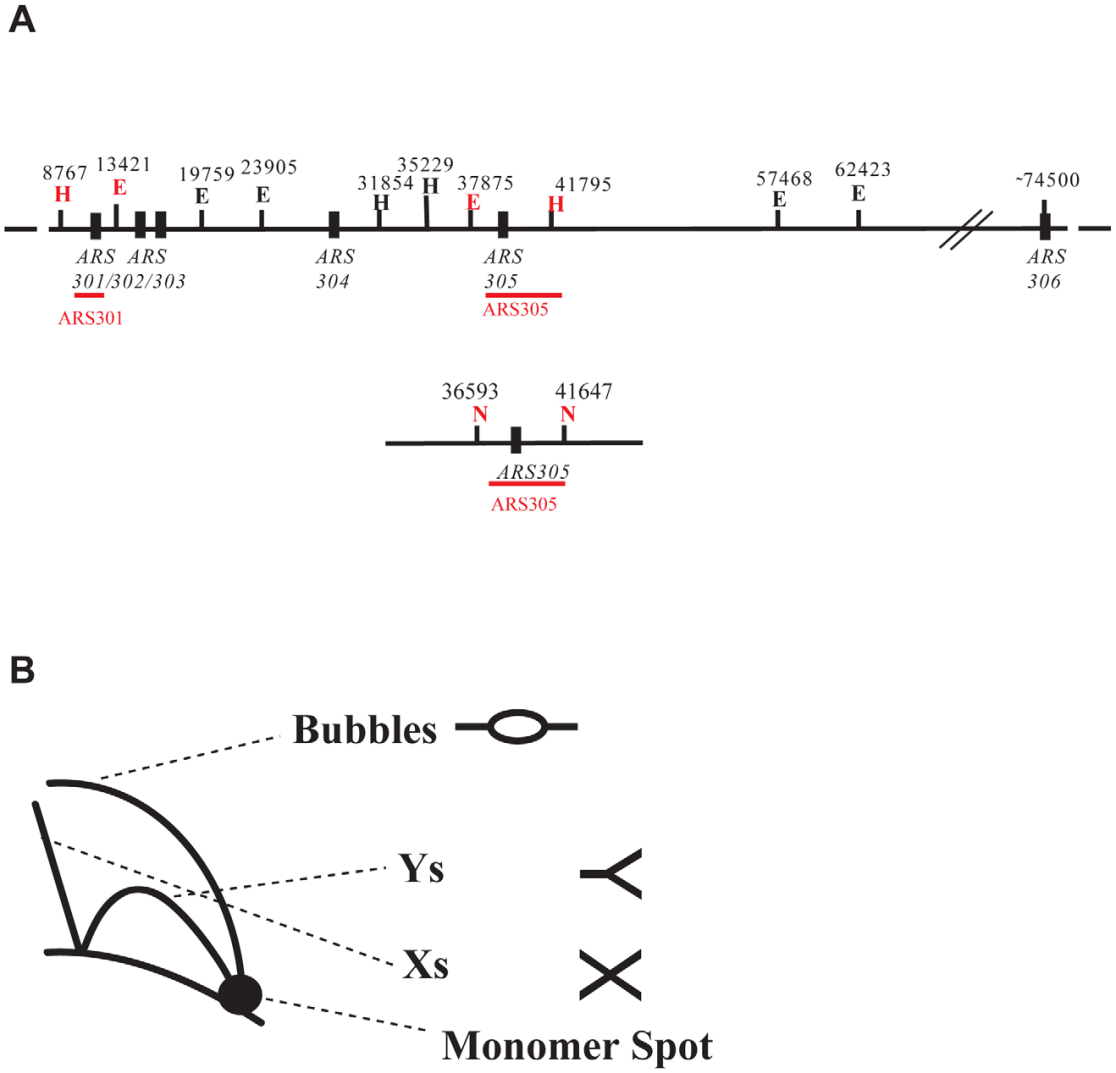
Schematic representation of 2D gel replication intermediates and genomic maps. (A) The genomic region containing the *ARS305* origin and the flanking regions on chromosome III. E and H stand for *Eco*RV and *Hind*III, respectively. N stands for *N*coI. The ARS305 probe spans from 39026 to 41647, the ARS301 probe from 10135 to 11416. (B) Schematic representation of the replication intermediates visualized by 2D gel electrophoresis.

Template switch events have been proposed to lead to the formation of SCJs in the proximity of damaged replication forks [10], [17]. To define the factors that affect the efficiency of template switch, we used 2D gel electrophoresis to analyze the profile of replication intermediates formed at an early efficient origin of replication located on chromosome III in *S. cerevisiae*, *ARS305*, and its flanking regions (Figure 1) [13]. In this assay, synchronized yeast cells are released and allowed to undergo the following S phase in a medium containing the alkylating reagent methyl-methanesulfonate (MMS). The pattern of replication intermediates is analyzed at different time points during replication.

Previous results have shown that Rad51-dependent X-shaped intermediates sharing the properties of pseudo-double HJs form during replication of damaged templates and accumulate in mutants affecting the functionality of the Sgs1-Top3 complex [10], most likely due to their impaired resolution [14] (Figure S1). Such molecules also form in wild-type cells, but are transient and scarce [10], [13], [24]. In order to facilitate our analysis of the contribution of different factors to the formation of the X-structures during replication of damaged templates, we took advantage of the *sgs1* mutant background and compared the amount of X-molecules formed in *sgs1Δ* with those formed in double mutants of *sgs1* and different repair genes.

### The role of the HR factors Rad55 and Rad59 in the formation of template switch intermediates

It has been demonstrated that Rad51 and Rad52 are required for template switch events leading to replication-associated SCJs in the proximity of replication forks [10], [16]. Whether the function of Rad51 in this process is related to its ability to stabilize the X-structures, which could be achieved by binding of Rad51 to the ssDNA stretches of the hemicatenane-like intermediates and formation of paranemic junctions (Figure S1) or of plectonemic DNA structures if one of the ssDNA strands is nicked, or rather to its active role in the formation of the structures, (e.g. by promoting strand invasion as in typical HR reactions) is not known. We examined the requirement of factors differentially affecting HR and strand exchange (RPA, Rad55, and Rad59) for template switch.

**Figure 2:**
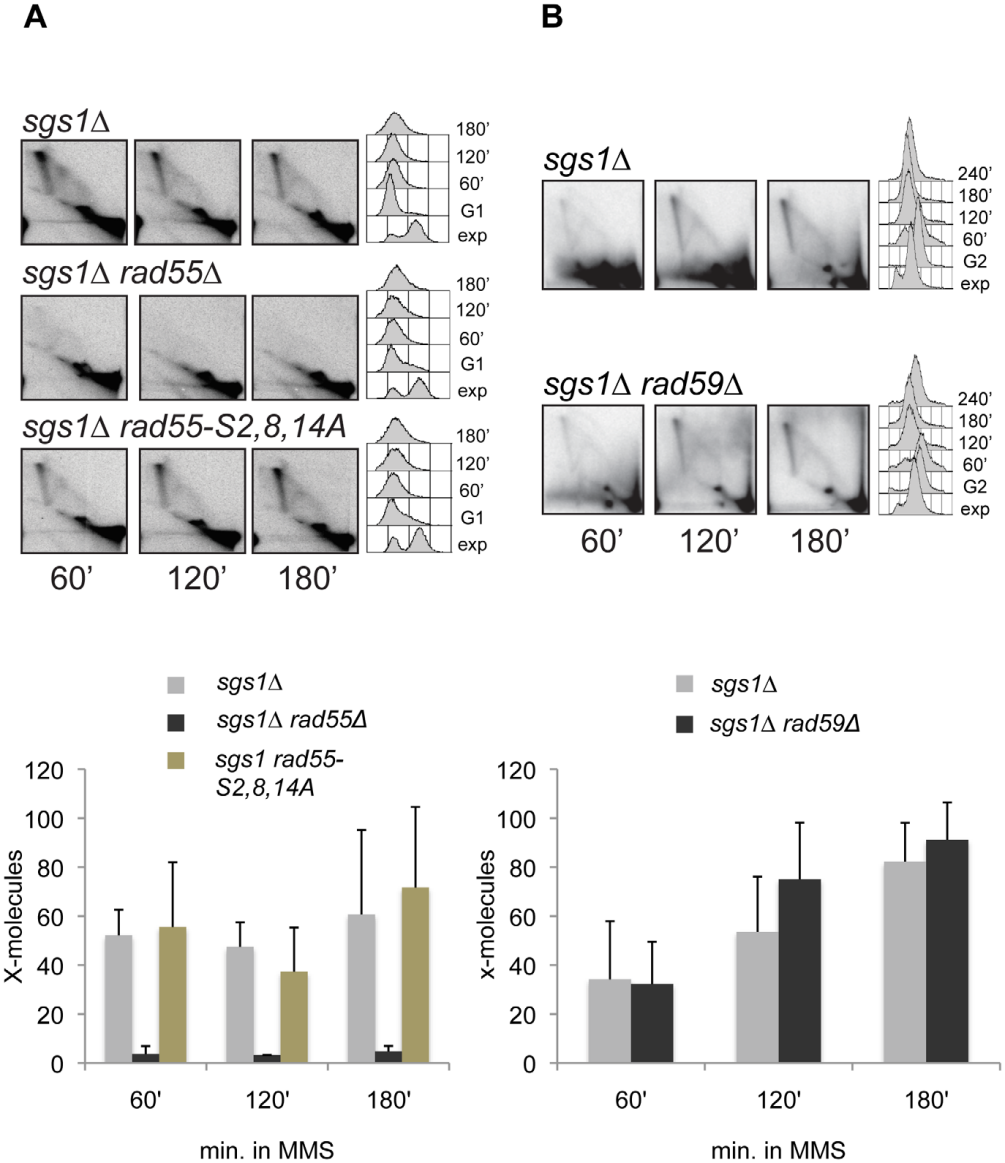
Rad55, but not Rad55 phosphorylation by Rad53 or Rad59, is required for template switch replication. (A) *sgs1Δ* (HY1465), *sgs1Δ rad55Δ* (HY1460) and *sgs1Δ rad55-S2,8,14A* (HY0799) and (B) *sgs1Δ* (FY1058) and *sgs1Δ rad59Δ* (HY1414) were arrested in G1 with α-factor (A) or with nocodazole in G2 (B) and released in medium containing MMS 0.033% at 30°C. At the indicated time-points samples were taken, the genomic DNA was extracted and digested with *Eco*RV and *Hind*III and the replication intermediates were analyzed by 2D gel with a probe recognizing the *ARS305* region.

Ablation of Rad55, known to have mediator functions [28], had an effect similar to that previously reported for *RAD51* and *RAD52* deletions [10], [16], abolishing the X-structures accumulating in the proximity of damaged replication forks in *sgs1*Δ (Figure 2A). We note that in the graphs showing the quantification of the X-structure, the % of spike represents a normalized value to the maximum amount of X-molecules observed during the time course rather than the % of total replication intermediates (see Materials and Methods for a detailed description of how quantification was performed). Although the mammalian orthologues of Rad55-Rad57 may also be implicated in late recombination events and/or resolution of recombination intermediates [35], [36], *rad55* single mutants (*SGS1*+ *rad55Δ* cells) behaved similarly to wild-type cells in this assay (Figure S2A).

The replication and damage checkpoint Rad53 is required for the formation of the X-structure [10] and also affects HR, but its substrates involved in regulating HR remain unknown (reviewed in [2], [7]). Phosphorylation of Rad55 by the Rad53 checkpoint kinase was reported to be important for damage tolerance under conditions of genotoxic stress perhaps by promoting recombinational repair [38]. We analyzed the effect of the *rad55* mutant in which the serines (Ser, S) 2, 8, and 14 phosphorylated by Rad53 were mutated to alanine (Ala, A) residues [38]. Unlike the *RAD55* deletion, the *rad55* phosphomutant did not affect the efficiency of template switch intermediates (Figure 2A and see Figure S2B), suggesting that Rad55 phosphorylation by the replication checkpoint is not essential for this process. Differently from *RAD55* and *RAD51* deletions, ablation of *RAD59* did not affect the X-molecule formation (Figure 2B and see Figure S2B). Thus, the Rad55-Rad57 mediator of HR is required also for template switch, but the crucial substrate of Rad53 in this process is not Rad55.

### The effect of the RPA mutation, *rfa1-t11*, on template switch

To further examine the role of recombination mediators and possibly of the checkpoint response in this process, we examined the effect of mutations in the ssDNA binding protein RPA *rfa1-t11* (K45E), in template switch. Although RPA can exclude recombinases from HR substrates and therefore has an inhibitory role in the assembly of the presynaptic filament and strand exchange [28], the *rfa1-t11* mutation in the largest subunit of RPA is associated with a synapsis defect [59], [60], suggesting that RPA plays a role in DNA strand invasion during HR. Biochemical characterization of RPA containing the mutant Rpa1-K45E subunit showed it to be inefficient in Rad51-mediated strand exchange [60]. Consistent with this report, other studies have also found *rfa1-t11* to be defective in recombinational repair [60]–[62]. We addressed whether *rfa1-t11* impacts the accumulation of X-molecules in *sgs1* mutants.

**Figure 3:**
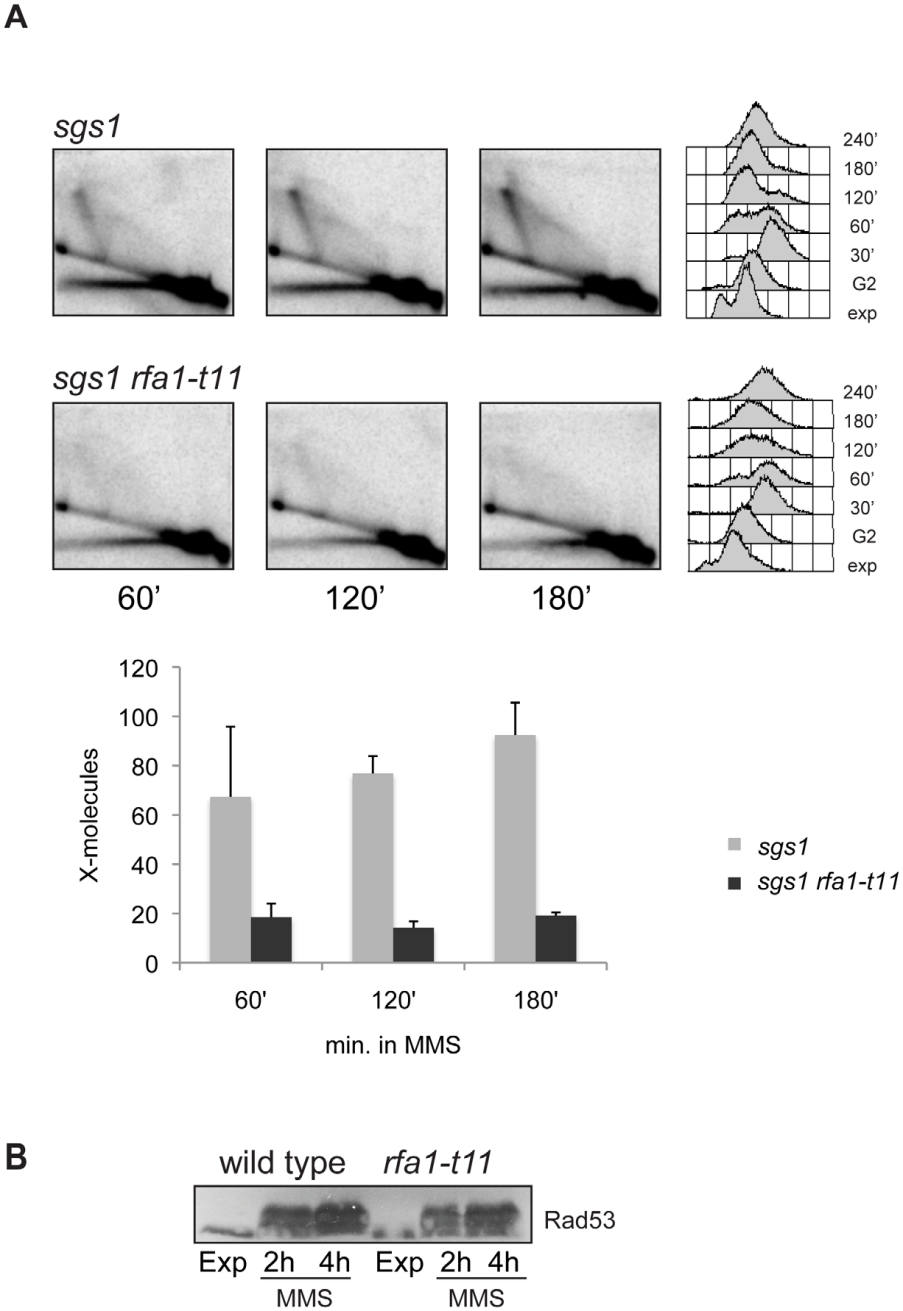
RPA, promoting the strand invasion step of homologous recombination, is required for template switch replication. (A) *sgs1* (HY1461) *and sgs1 rfa1-t11* (HY1459) were synchronized in G2 with nocodazole and released in medium containing MMS 0.033% at 28°C. The replication intermediates were digested with *Nco*I and analyzed with the ARS305 probe. (B) Exponentially growing wild-type (W303-1A) and *rfa1-t11* (HY1464) cells were treated for 2 and 4 hours with MMS 0.02%. Western blot analysis was performed to detect Rad53 phosphorylation.

As *sgs1Δ* was reported to be synthetic lethal or have severe growth defects with many mutants affecting replication and/or recombination [63]–[65], we utilized a hypomorphic *sgs1* mutant in which the helicase activity is impaired due to the insertion of the *AUR1-C* marker in the helicase domain of Sgs1 but which has milder phenotypes than *sgs1Δ* [66]. The *sgs1::AUR1-C* mutant was shown to accumulate X-molecules during replication of damaged templates [13], [17], in line with findings reported for other *sgs1* alleles affecting the helicase activity of Sgs1 [67]. The *rfa1-t11* mutation significantly decreased the accumulation of X-molecules in *sgs1* (Figure 3A). The original report on *rfa1-t11* showed it to be proficient in DNA replication [62]. In agreement with this view, we also find that under conditions of DNA damage the profile of replication intermediates is not affected by the *rfa1-t11* mutation at *ARS305* or the flanking region *ARS301* (Figure S3 and data not shown). Thus, the effect of *rfa1-t11* on the X-molecules formed in the proximity of replication forks cannot be attributed to general replication problems.

Since RPA bound to single-stranded (ss) DNA is a signal for Rad53 checkpoint activation ([7] and references therein), and Rad53 is required for the template switch X-formation [10], it was important to establish whether the defects observed for *rfa1-t11* in X-molecule formation under conditions of DNA damage are due to strand exchange defects and/or inability to boost Rad53 activation. The reports on the role of *rfa1-t11* in checkpoint response are controversial: some studies found it defective for the replication/damage checkpoint [68]–[71], while others found it proficient [62], [72]. We found no evidence for impaired Rad53 activation in *rfa1-t11* mutants either in spontaneous or MMS-treated conditions (Figure 3B), suggesting that its effect in this context is more related to recombination and strand-exchange rather than checkpoint signaling. This result also allows us to conclude that the gaps formed during replication can still elicit a robust checkpoint response, mediated by RPA, in the absence of X-molecule formation.

### The Exo1 exonuclease is required for efficient damage-induced template switch events

Exo1 is a member of the Rad2 family of structure-specific nucleases and possesses a 5′-3′ exonuclease activity ([73], [74] and references therein). Exo1 was implicated in processing abnormal structures arising at stalled replication forks [75], [76], in the checkpoint response [77], DSB resection [78]–[80], and other DNA repair events including mismatch and post-replication repair (PRR) (reviewed in [74]).

**Figure 4:**
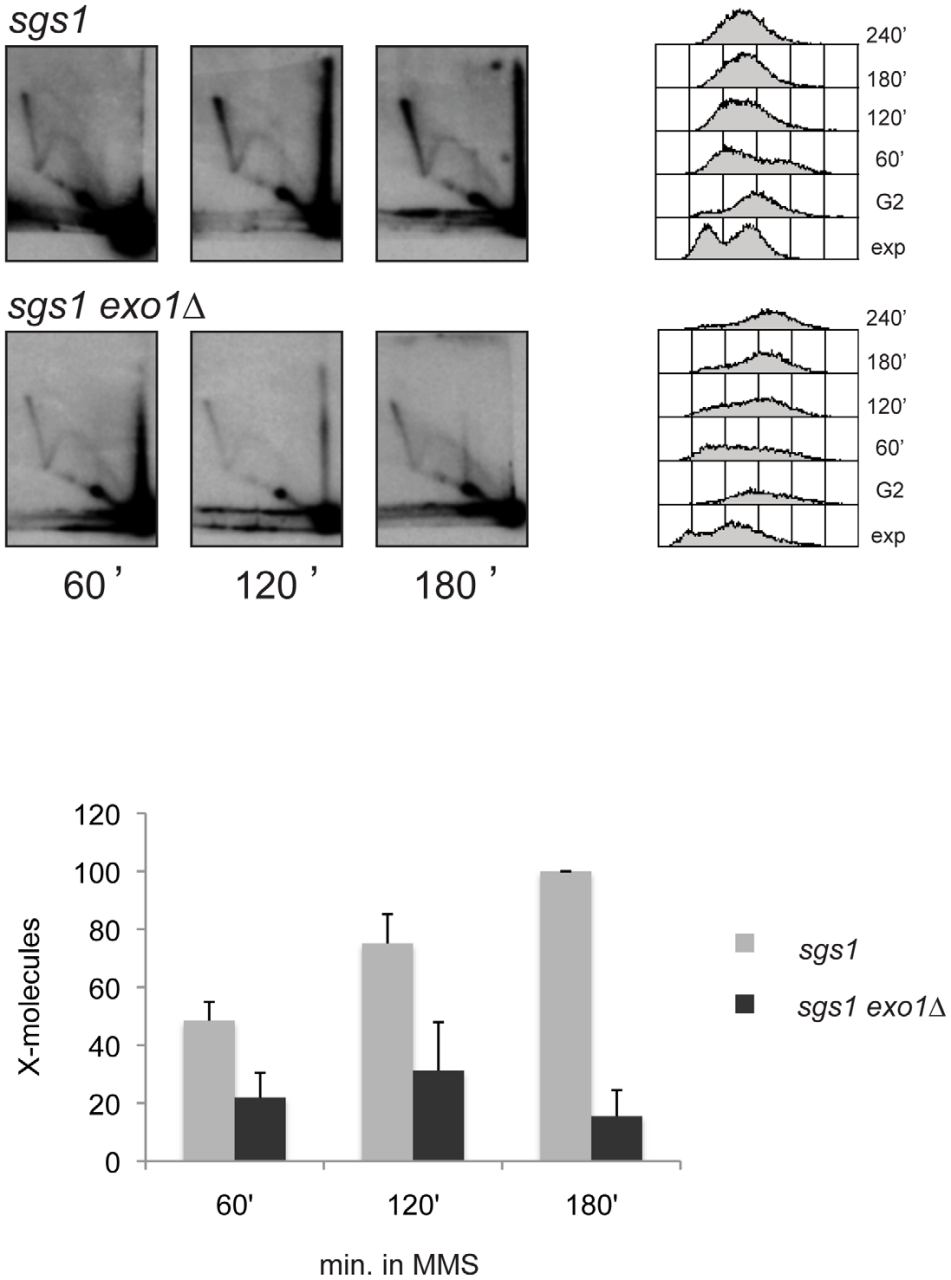
Exo1 contributes to damage-bypass replication by template switch. 2D gel analysis of replication intermediates digested with *Nco*I from *sgs1* (HY1461) and *sgs1 exo1Δ* (HY1448) cells synchronized with nocodazole and released in medium containing MMS 0.033% at 28°C.

Here we addressed the involvement of Exo1 in the formation of SCJ molecules during replication of damaged templates. A combination of *exo1Δ* and *sgs1Δ* mutations leads to a severe growth defect, in accordance with previously published reports [63]. In attempts to overcome the cell-cycle delay and the general genome instability often associated with severe growth defects, we used the truncated *sgs1* mutant described above and in previous works [66]. The *sgs1 exo1Δ* double mutant combination was still growing slowly in comparison with each single mutant, but the growth was not as severely affected as in *sgs1Δ exo1Δ* cells. We found that *exo1Δ s*ignificantly reduced the amount of X-molecules accumulating in *sgs1* mutants (Figure 4). The single mutant *exo1* had a similar pattern of replication intermediates compared to wild-type cells (Figure S4).

### TLS polymerases are not required for template switch–mediated damage bypass

**Figure 5:**
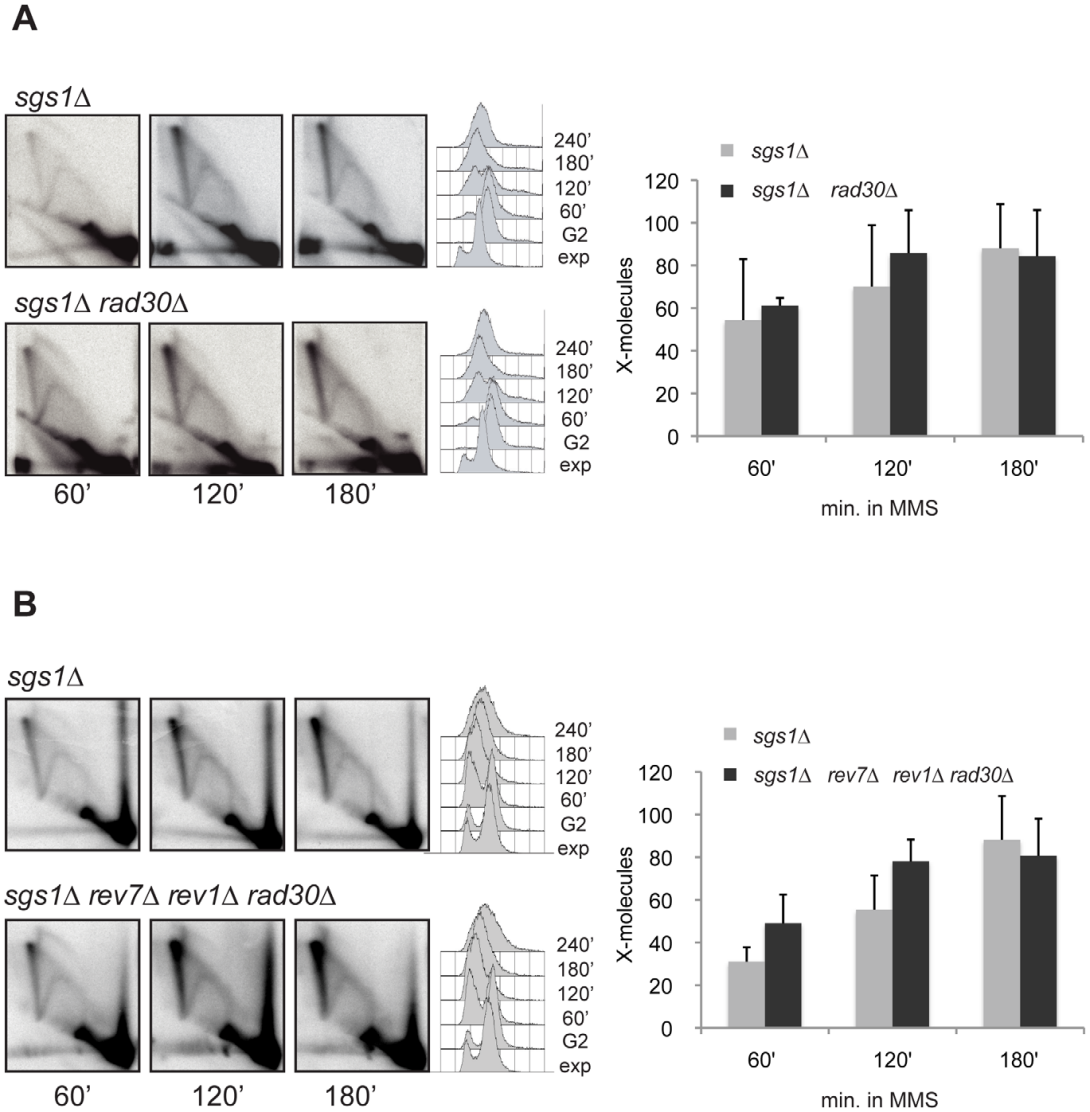
Translesion synthesis polymerases do not contribute to the DNA synthesis step of template switch. (A) *sgs1Δ* (HY1465) and *sgs1Δ rad30Δ* (HY1467) cells and (B) *sgs1Δ* (HY1465) and *sgs1Δ rev7Δ rev1Δ rad30Δ* (HY1468) cells were synchronized in G2 with nocodazole and then released in YPD medium containing MMS 0.033%. Both experiments were performed at 28°C and samples were taken for 2D gel analysis; the DNA was digested with *Eco*RV and *Hind*III and the membrane hybridized with a probe corresponding to *ARS305*.

We addressed the possibility that specialized polymerases may be required for the DNA synthesis step of the template switch process. Mutations in DNA polymerases often sensitize cells to DNA damage, including MMS (Figure S5), but since this could reflect defects of these mutants in various DDT or repair pathways, it is hard to infer based on this sensitivity spectrum the contribution of the different polymerases to template switch. Previous work has shown that Polη can efficiently extend artificial D-loop substrates [57] and that chicken Polη affects Ig gene conversion tracts [58]. We thus analyzed the role of Polη, encoded by the *RAD30* gene in yeast, in the formation of the X-structures accumulating in *sgs1*Δ, but observed no significant decrease in *sgs1*Δ *rad30*Δ mutants (Figure 5A). In addition to Polη, other specialized TLS polymerases can facilitate damage-bypass; in budding yeast they are Polζ (composed of the Rev3 catalytic subunit and the Rev7 non-catalytic subunit) and Rev1, which functions mostly in conjunction with Polζ but may also act to mediate the switching between TLS polymerases specialized for insertion and those required for extension [1], [4]. We found that ablation of Polζ by *REV7* deletion, or concomitant inactivation of Polζ and Rev1 (*rev7*Δ *rev1*Δ), or of all TLS polymerases in yeast (*rev7*Δ *rev1*Δ *rad30*Δ) did not reduce the X-molecule accumulation in *sgs1*Δ cells (Figure 5B and data not shown), suggesting that TLS polymerases do not play a major role in the DNA synthesis step required for template switch repair. The TLS mutants in a wild-type (*SGS1*+) context did not affect the pattern of replication intermediates (Figure S6). We also note that this result does not imply that translesion synthesis is less important than template switch in DDT, as in our system TLS-mediated lesion bypass events not involving X-molecules are not detected.

### Differential requirements for replicative polymerases in template switch replication

**Figure 6:**
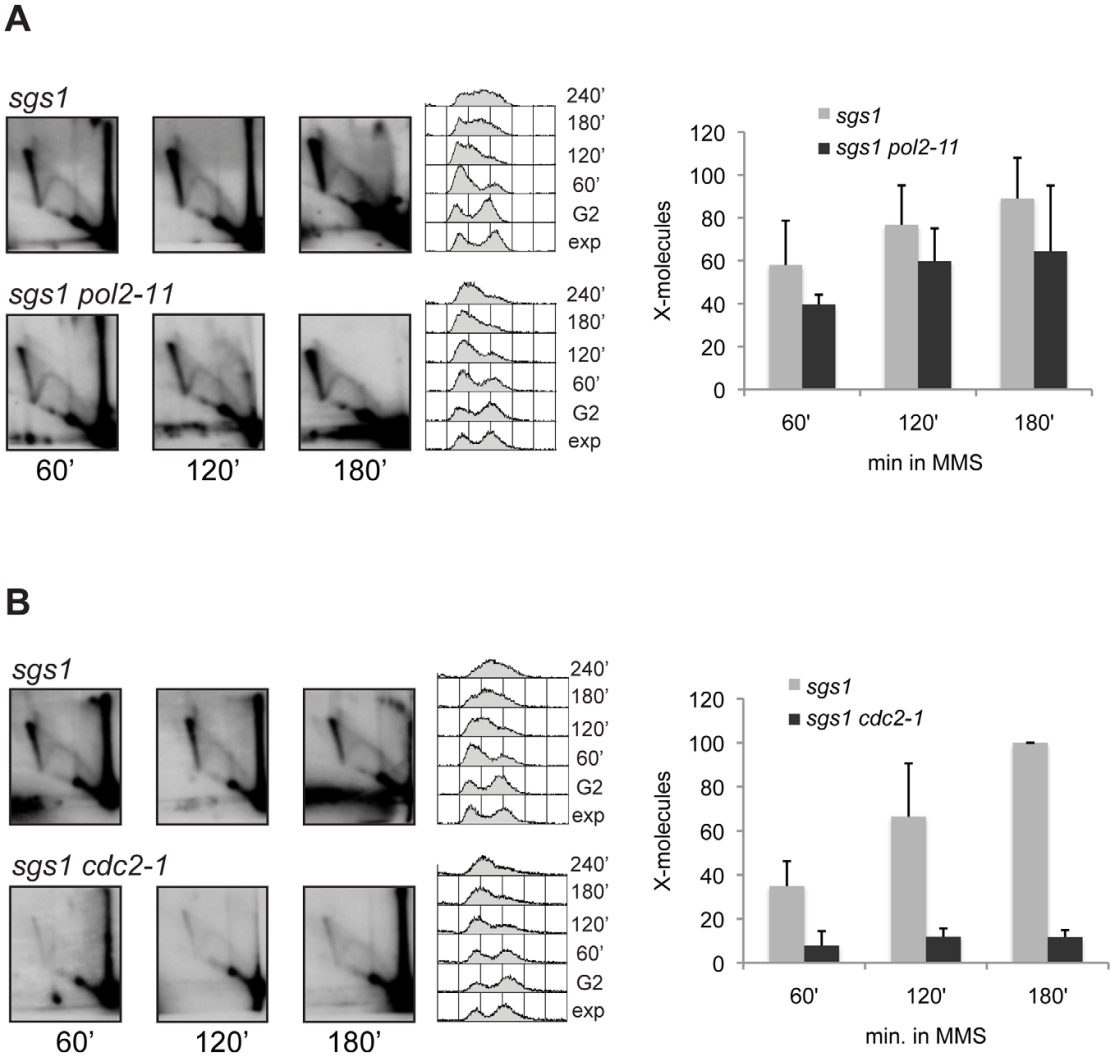
Polδ but not Polε, is required for template switch replication. 2D gel analysis of replication intermediates in (A) *sgs1* (HY1455), *sgs1 pol2-11* (HY1456) and (B) *sgs1* (HY0100), *sgs1 cdc2-1* (HY0103). The experiments were performed at the semi-permissive temperature of 30°C. The DNA samples were digested with *Hin*dIII and *Eco*RV and the membranes hybridized with a probe corresponding to *ARS305*.

We also addressed the contribution of the main DNA polymerases required for elongation during eukaryotic genome replication: Polε and Polδ. We first examined by FACS the temperature at which these polymerase mutants do not impair cell cycle progression and found that at 30°C the Polδ mutant, *cdc2-1*, and the Polε mutant, *pol2-11*, are able to complete replication, while at 37°C these cells have a prominent delay in S-phase progression (data not shown and see Figure S7). This finding is in accordance with previous reports showing that *cdc2-1* mutants fail to replicate approximately one third of their nuclear genome at restrictive temperatures [81], [82]. To minimize the general replication defects inherently associated with Polδ and Polε mutations, we used permissive conditions of replication (30°C) and analyzed the effect of these polymerase mutants on the X-molecules forming in the proximity of early origins of replications (*ARS305*), which are less prone to replication delays/problems as compared to later replication zones. The double mutants between *sgs1* and either *pol2-11* or *cdc2-1* were viable, although *sgs1* mutation induced lower viability of *cdc2-1* cells at 30°C and increased the percentage of cells in G2/M under normal growing conditions (Figure S7). The *cdc2-1* mutation in Polδ drastically diminished the amount of X-molecules in *sgs1Δ*, whereas *sgs1 pol2-11* cells accumulated a similar amount of X-structures with *sgs1* (Figure 6). The polymerase mutants, *pol2-11* and *cdc2-1*, in a wild-type (*SGS1*+) context did not affect the pattern of recombination-like X-intermediates (Figure S8).

To further establish that the effects of the *cdc2-1* mutation on X-molecules are not due to general replication problems as opposed to a requirement for Polδ in template switch DNA synthesis, we attempted to gauge the differential effects of *cdc2-1* in replication versus X-structure formation. For this purpose, we quantified the effect of *cdc2-1* on both Y arcs (representing replication forks) and X-molecules. The results indicate that the reduction in the Y signal at *ARS305* caused by the *cdc2-1* mutation is much lower in magnitude than its effect on the X-molecules; accordingly the ratio of X-molecules versus Y arcs, which represents the amount of X-molecules normalized to the ongoing replication in the analyzed genomic fragment, is much lower in *sgs1 cdc2-1* as compared to *sgs1* (Figure S9). To further examine the effect of *cdc2-1* on X-molecules versus DNA replication, we have also followed the progression of the forks to the *ARS301* region (Figure S10), which is replicated passively by forks coming from *ARS305* (see Figure 1A). The progression of the replication forks in this region in a *cdc2-1* background showed kinetics similar to those observed in *SGS1+* cells. Notably, at all regions and time points analyzed the effect of *cdc2-1* mutation on the X-signal was much more profound than its effect on the Y molecules (Figures S9 and S10).

**Figure 7:**
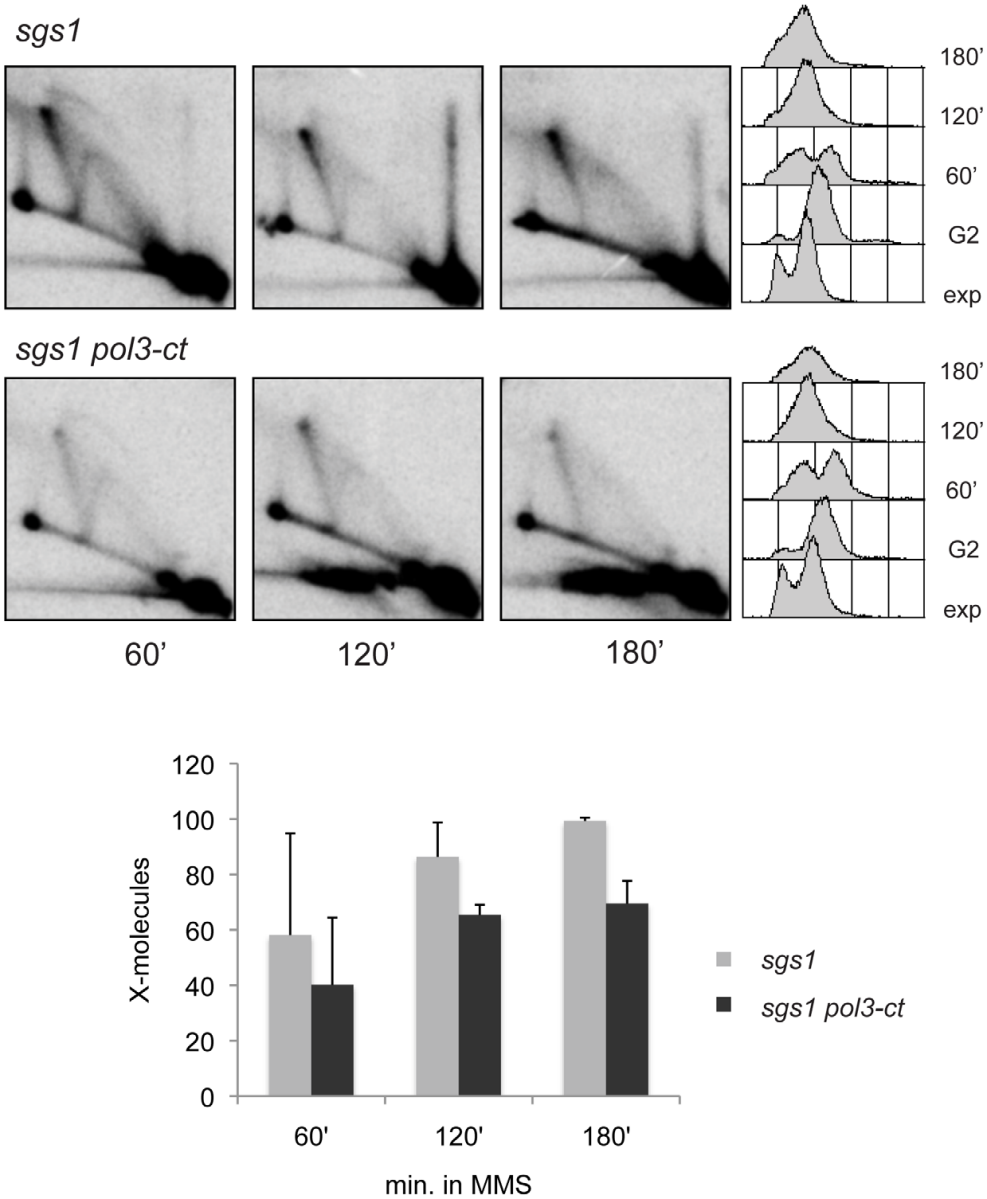
The effect of the replication-proficient *pol3-ct* mutation in template switch replication. 2D gel analysis of replication intermediates forming at *ARS305* from *sgs1* (HY1461) and *sgs1 pol3-ct* (HY1257) cells replicating in the presence of MMS damage at the permissive temperature of 30°C. The p-values obtained from unpaired t-tests for 120 min (P = 0. 0089) and 180 min (P = 0. 0022) indicate that the differences between *sgs1* and *sgs1 pol3-ct* are statistically significant.

To further test the role of Polδ in template switch we examined the effects of the *pol3-ct* mutant, reported not to have defects in DNA replication [83]. We observed that the replication kinetics of *pol3-ct* as assessed by FACS are identical to that of wild-type (Figure 7 and Figure S11). When *pol3-ct* cells were crossed with *sgs1*, we easily obtained *pol3-ct sgs1* double mutants, and their doubling time at 30°C was similar to that observed for *sgs1* single mutant (*pol3-ct*: 90′±5′; *sgs1*: 100′±4′; *pol3-ct sgs1*: 100′±8′). We found that the *pol3-ct* mutation reduces the amount of X-molecules accumulating in *sgs1* to about 70%; although small, this effect was highly reproducible (Figure 7). The *pol3-ct* mutant had a similar pattern of replication intermediates with wild-type (Figure S11).

**Figure 8:**
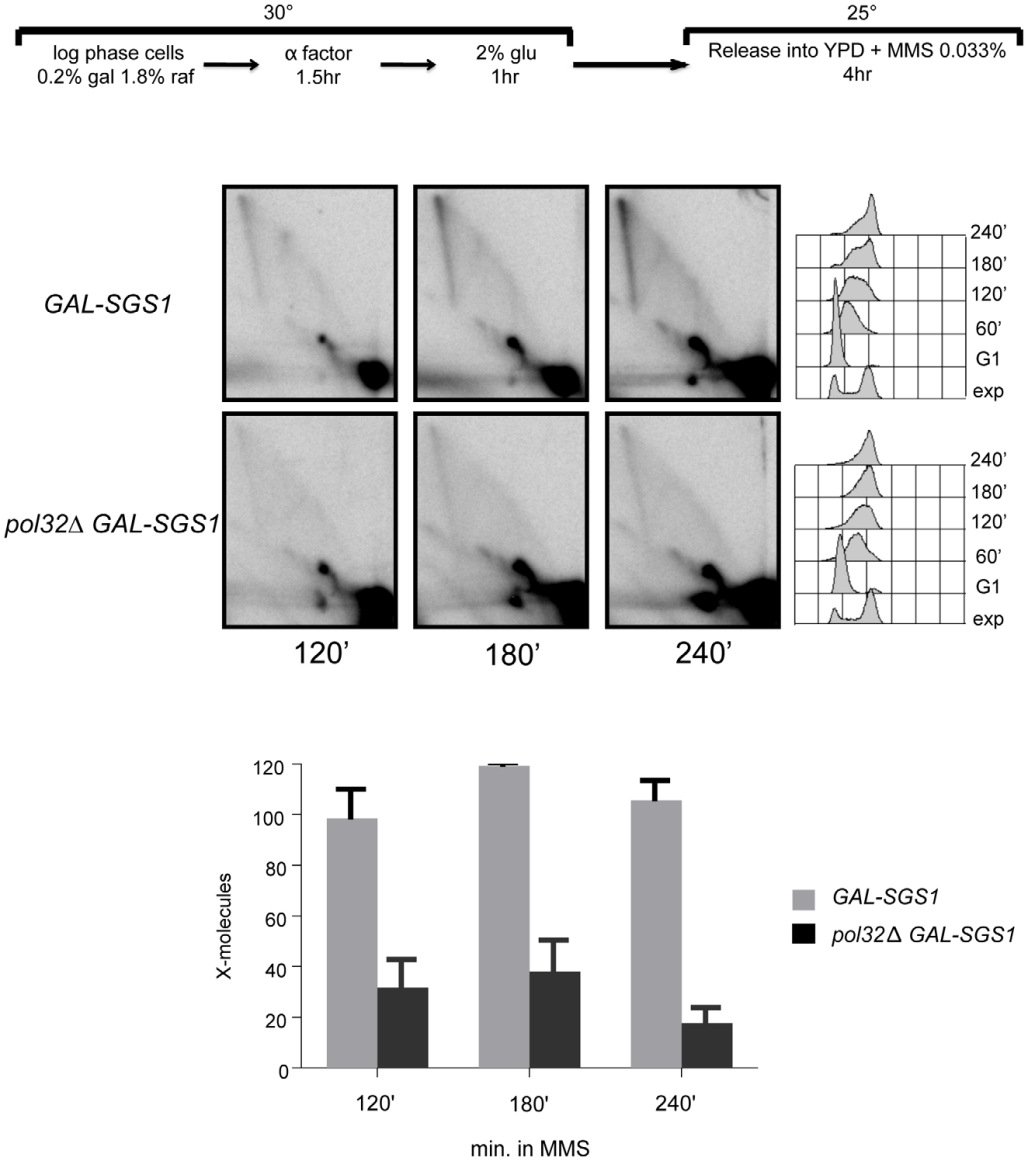
The effect of the *pol32*Δ mutation in template switch replication. 2D gel analysis of replication intermediates from *GAL-SGS1* (FY1359) and *GAL-SGS1 pol32*Δ (FY1379) under conditions in which *SGS1* expression was shut-down by the addition of glucose. The cells were grown to log phase in YP-media containing raffinose and galactose at 30°C, arrested in G1 and then released in YPD containing MMS 0.033% at 25°C for 4 hours. The DNA samples were digested with *Hin*dIII and *Eco*RV and the membranes hybridized with a probe corresponding to *ARS305*.

Considering that *sgs1 cdc2-1* is more slow-growing than *cdc2-1* likely due to the accumulation of spontaneous lesions (see Figure S7), and *pol3-ct* has a minor effect on the X-accumulation (Figure 7), we decided to further analyze the effect of mutating the third, non-catalytic subunit of Polδ, Pol32, known to affect the processivity of the Polδ complex [84], [85]. The combination of *pol32* and *sgs1* mutations leads to marked slow growth at 28°C and 30°C, and lethality at lower temperatures, such as 23°C, which is still permissive for *pol32* [11]. To override the undesirable effects on replication caused by the delayed growth of the double mutants, we employed a conditional *SGS1* system, *GAL-SGS1*, in combination with the *pol32* mutation, previously reported [11], in which *SGS1* shut-down is induced only during the course of the experiment, by addition of glucose (Figure 8). The *pol32* mutation had a clear effect in reducing the X-accumulation under such experimental settings. Notably, in these conditions the progression through S-phase of the double mutant or of the *pol32* single mutant did not appear to be impaired (Figure 8 and Figure S12), in line with previous reports showing that the problems experienced by *sgs1 pol32* cells and leading to low-viability are caused by G2 events [11].

Altogether, these last sets of results suggest that Polδ plays an important role in mediating the DNA synthesis step of template switch. Since Polδ is also required for bulk replication, and most template switch are likely to occur behind replication forks [11]–[13], our results imply that Polδ acts in a distributive manner, both at the fork, to promote DNA replication, and behind the fork, to promote post-replicative repair events such as template switch.

## Discussion

**Figure 9:**
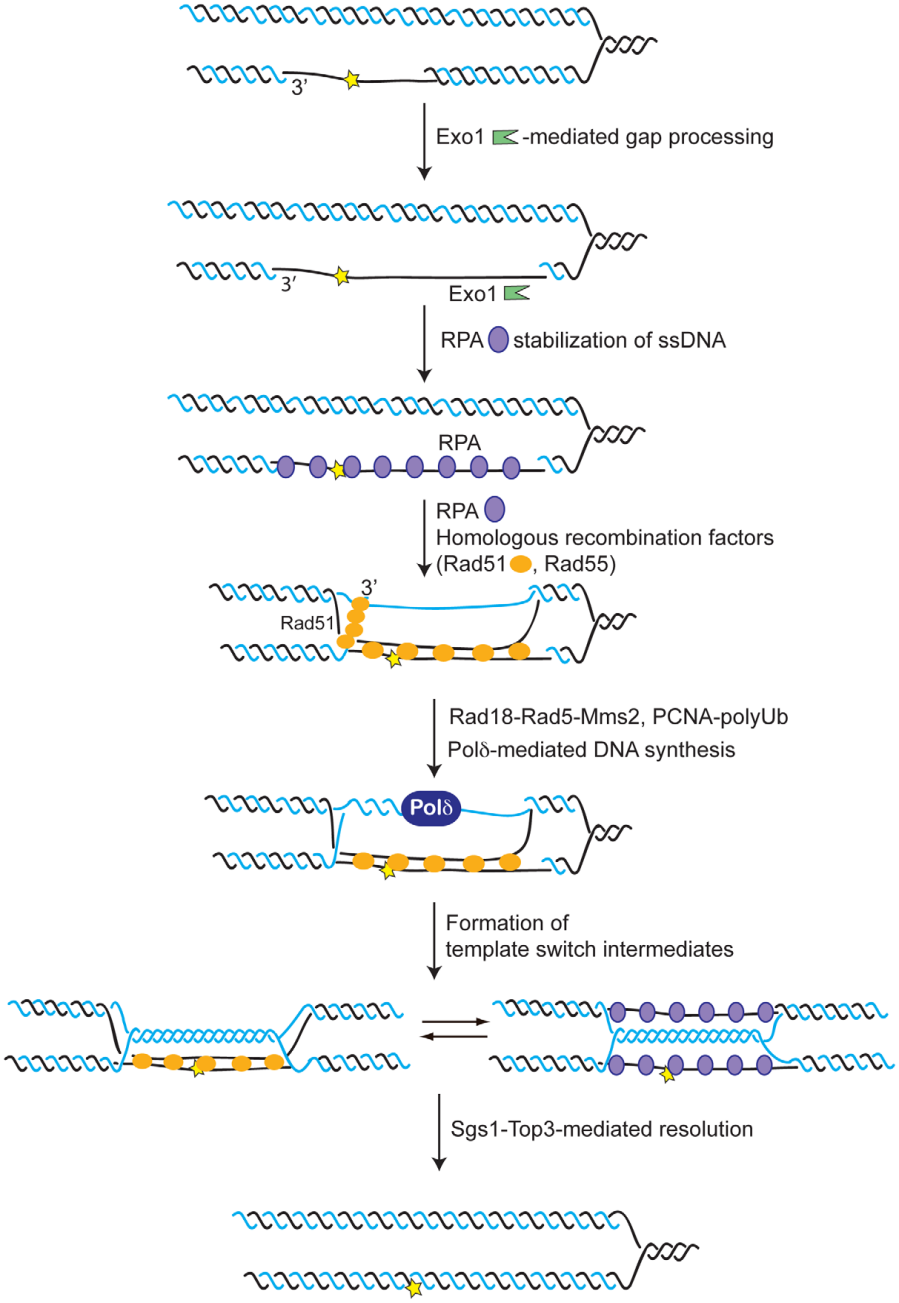
Model for factors contributing to template switch replication. DNA damage during replication can cause uncoupling between the leading and lagging strands. In this model the DNA lesion is represented on the leading strand behind the replication fork. The gaps are further processed by the Exo1 nuclease to expose ssDNA. RPA coats ssDNA to stimulate recombination events. Additional recombination factors including Rad52, Rad51, and Rad55-Rad57 promote strand invasion into the homologous duplex. The newly synthesized sister chromatid serves as a template for DNA synthesis, which is mediated mainly by Polδ. The activities regulating Polδ-loading and Polδ-mediated DNA repair synthesis remain to be investigated; they may involve Rad18-Rad5-Mms2 functions and PCNA modifications. The 3′end of the nascent invading strand returns to its parental template, giving rise to the X-shaped template switch intermediates. The possibility that Rad51 stabilizes the ssDNA stretches of the X-intermediates into paranemic junctions is also represented. The template switch intermediates are then resolved by the action of Sgs1-Top3.

Template switch, thought to be implicated in both gap-filling and restart of replication forks stalled by DNA lesions, plays an important role in DNA metabolism and may protect against chromosomal instability via stabilizing repetitive sequences, preventing translocations and instability associated with certain genomic disorders [2], [86]. The mechanism and genetic factors promoting or controlling template switch are not well understood. The goal of our present study was to deepen our understanding of how template switch occurs and is regulated. To this purpose, we addressed the contribution of different factors to the formation of replication-associated SCJs, thought to represent template switch intermediates [5], [6], [10], [13]. Several observations concur with the idea that template switch occurs mainly behind replication forks to fill in the ssDNA gaps accumulating under damaging conditions [11]–[13] (Figure 9). We do not exclude the possibility that a fraction of template switch events may occur at the site of lesion via other DNA intermediates such as reversed forks; future studies will be needed to both elucidate the proportion of bypass events occurring at the site of the lesion as well as to understand whether alternate pathways leading to regressed fork formation are subjected to regulation in the cells exposed to genotoxic stress.

Besides alkylating bases, MMS may cause DSBs, although this notion remains controversial [87], [88]. To what degree the factors implicated in DSB repair are required for replication-associated template switch and other SCR events is not known and thus, automatic extension of the existing genetic data aimed at elucidating the DSB response pathways to other recombination-like mechanisms involving HR factors, such as template switch, should be viewed cautiously. HR is most active in S and G2/M phases of the cell cycle, but it is likely that different lesions or DNA substrates will involve distinct crosstalks between repair proteins and cell-cycle or damage response signaling pathways in order to promote DDT [2], [89]. Furthermore, whether DSB repair pathways operate primarily in S-phase to restart forks or in G2 to promote replication completion and DNA repair, remains an issue of debate. Considering that the X-structures generated during replication under conditions of MMS damage do not represent canonical Holliday Junctions, and furthermore, that Sgs1-Top3, and not Holliday Junction nucleases, represent the main activities required for the X-resolution in S-phase [10], [18], it seems logical to assume that the template switch X-structures formed in the proximity of replication forks require distinct sets of factors and are, at least partly, different from the DNA intermediates generated during other DSB repair processes. On the other hand, it is also reasonable to foresee that the pathways implicated in replication-associated HR-mediated DSB repair (BIR) and template switch-mediated gap filling will share common enzymatic activities. In this study we have uncovered both similarities (Rad51, Rad55-Rad57, Rfa1, Polδ) and dissimilarities (Polε, Rad59) between the factors implicated in these two processes (see also below), but future studies will be needed to deepen our understanding of the mechanisms through which different recombination-mediated pathways are coordinated with one another and other cell-cycle signaling networks to promote damage avoidance.

By using 2D gel analysis of replication intermediates and a genetic set-up in which the template switch DNA structures are enriched by preventing their resolution mediated by Sgs1-Top3 [10], [13], we established here that, in addition to factors known to be required also for the strand invasion step of HR, Polδ plays an important role in the efficient formation of template switch intermediates. Previous genetic studies have also defined a role for Polδ in gap and DSB repair [46], [90]–[93]. While the *pol2-11* mutation affecting Polε function was previously reported to affect the DNA synthesis step of break-induced replication (BIR) [46], it did not significantly affect the formation efficiency of forming template switch intermediates in the proximity of replication forks (Figure 6A), suggesting that differences exist even at the elongation step between different recombinational repair pathways activated by replication problems.

The *cdc2-1* allele in the catalytic subunit of Polδ, previously shown to be defective in the repair of MMS-induced single-strand breaks at non-permissive temperatures [94], [95], also affects the efficiency of template switch intermediates at temperatures that are permissive for replication (Figure 6B). The observation that *cdc2-1* cells are able to establish forks even at late replication zones under conditions permissive for growth with kinetics similar to those observed in wild-type cells (Figure 6 and Figure S10) compellingly suggests that the defect of *cdc2-1* in X-formation reflects a bona-fide role of Polδ in template switch DNA synthesis (Figure 8) and that this defect cannot be fully attributable to the replication defects of *cdc2-1* cells [81], [82] (Figure 6 and Figure S9). Work done on other alleles of Polδ, such as the *pol3-ct* allele having a truncation that removes the last four amino acids of the Pol3 protein, has uncovered a role for Polδ in HR-mediated DNA synthesis during gene conversion (GC) [93], a DSB repair pathway occurring when homology with both the DSB ends is present, as well as in BIR [92]. In our system, we find that *pol3-ct* had a small but reproducible effect on the efficiency of forming template switch intermediates (Figure 7). Furthermore, by using a conditional *sgs1* system in combination with a null mutation in the non-essential subunit of Polδ, Pol32, we were able to establish that impairment of Polδ function and processivity by the *pol32Δ* mutation, largely impacts on the ability of cells to undergo template switch (Figure 8).

Specialized TLS polymerases may also contribute to the DNA synthesis step of template switch. Although Polη can extend the invading 3′end of a D-loop intermediate in vitro and deletion of chicken Polη reduces the frequency of DSB-induced gene conversions [57], [58], we could not assign a significant role for Polη or other TLS polymerases in the DNA synthesis step of template switch (Figure 5). However, given the biochemical data indicating that Polη could promote GC of up to about 80 nucleotides in vivo [58], while the gaps forming during replication of damaged templates in yeast have been estimated at approximately 400 nucleotides in size [12], it is possible that Polη plays a role, redundant with other polymerases such as Polδ, but which may therefore be too subtle to appreciate in our system. Nevertheless, we note that our results are in line with previous studies that have reported no role for Polη in the DNA synthesis steps occurring during GC or BIR in budding yeast [92], [93]. Our studies do not rule out a possible contribution of other DNA polymerases such as Polη and Polε to the DNA synthesis step of template switch; they do show, however, that TLS polymerases and Polε do not support wild-type levels of template switch when Polδ is inactive (Figure 9).

Since most template switch events are likely to occur in the rear of replication forks [11], [13], the emerging question is how the function of Polδ is divided to suit both its role in catalyzing highly processive replication at replication forks and its repair role in template switch. One line of control could be achieved by upregulating the amounts of DNA polymerases to greater levels than the ones strictly needed to perform DNA replication [96]; this could explain why limiting amounts of Polδ were shown to be associated with repair defects and chromosomal instability [97]. The functional versatility and local distribution of Polδ may be mediated by its interaction with different sets of proteins, such as those between Polδ and differently modified forms of PCNA (Figure 9). Indeed Rad18-Rad5-Mms2-mediated polyubiquitination of PCNA [98] promotes template switch [13] and stimulates the repair activity of Polδ [11], [99], [100]. Recruitment of Rad18 to RPA-coated ssDNA containing DNA lesions [101] may subsequently influence a number of processes required for efficient template switch such as the remodeling of PCNA through posttranslational modifications [98], the repair function of Polδ [11], [99], or the efficiency of HR per se [102] (Figure 9).

The replication checkpoint kinase Rad53 is implicated in template switch [10], but its role and targets in this process remain topics of investigation. Although strand exchange mediator activities, including that of Rad55, are crucial for the formation of template switch intermediates (Figure 2 and Figure S2), we found that direct phosphorylation of Rad55 by Rad53 [38] is not required in this specific context (Figure 2A). Gap extension before homology search can initiate is also expected to favor template switch; our results suggesting that Exo1 promotes template switch (Figure 4) classify it as a new factor of the error-free PRR and may reflect its role in processing the gaps formed behind replication forks under conditions of genotoxic stress (Figure 9). Extension of the gaps could be expected to lead to longer stretches of ssDNA-RPA and robust Rad53 activation; although we did not observe any obvious defect of *exo1* mutants in activating Rad53 following DNA damage (Figure S13), the effect may be too subtle or redundant with other nuclease-mediated pathways. Indeed, other nuclease activities, such as those of Sae2 and of the Mre11-Rad50-Xrs2 (MRX) complex have been shown to work together with Exo1 in other settings related to recombinational repair [74], [78]–[80]. Although we could not directly assess the contribution of these nucleases to template switch due to the severe growth defects of the nuclease double mutants with *sgs1*, we do not exclude the possibility that these three nucleases (Exo1, Sae2, Mre11) may all contribute independently or work with each other to promote template switch. Uncontrolled and extended gap processing – as seen in checkpoint mutants [12], [103] – should be avoided in order to preserve genome integrity, and if Exo1 is the key factor mediating these events [75], [76], it is reasonable to think that mechanisms exist to keep its activity under control. Indeed, recent studies have found that Rad53-dependent phosphorylation of Exo1 may limit ssDNA accumulation and act as a feedback to restrain checkpoint activation [77]. Since Rad53 phosphorylates Exo1 following MMS damage [77], [104], it is possible that the crosstalk between Exo1 and Rad53 is important for the regulation of template switch events as well as other mutagenic processes. Another possible role for Rad53 and the DNA damage checkpoint in template switch may be to favor or limit the strand invasion step or the processing of the recombination intermediates if the DNA synthesis step is hindered, for instance by controlling the activity of nucleases that may process the stalled/abortive recombination intermediates.

Future challenges will lie in characterizing how other players, including factors required for chromatin organization or sensing the topological status of the DNA, cooperate with repair and replication factors to modulate the division of labor between polymerases and to enable the functional versatility of proteins such as PCNA and Polδ. Finally, understanding how different DNA synthetic and repair demands are orchestrated to prevent the accumulation of DNA damage and maintain chromosomal stability has important implications for enhancing our knowledge of how cells are protected from cancer-causing alterations.

## Materials and Methods

### Yeast strains and plasmids

The yeast strains used in this study are mostly derivatives of W303 and the relevant genotypes are shown in Table S1.

### Growing conditions, cell cycle arrests, and drug treatments

Unless otherwise indicated, strains were grown at 28°C in YP-media containing glucose (2%), YPD, as carbon source, with the exception of experiments presented in Figure 8 and S12, where raffinose (1.8%) and galactose (0.2%) were used instead. Cells were synchronized either in metaphase by adding nocodazole to a final concentration of 10 mg/ml together with DMSO to a final of 1% v/v, for about 2.5 hr, or in G1 with α-factor to a final concentration of 3–4 µg/ml. The release from the synchronization was performed as previously described, in YPD containing MMS at a final concentration of 0.033% v/v [17], with the exception of the experiment in Figure S12, where the release was done in YP-media supplemented with raffinose (1.5%) and galactose (0.5%) and containing MMS 0.033%.

### Spot assays of drug sensitivity

Log phase cells were counted and 10-fold series dilutions were spotted on plates containing various concentrations of MMS and incubated at the indicated temperatures.

### Protein techniques

Western blot analysis and TCA extraction of yeast proteins was performed as previously described [18]. Rad53 was detected with the mouse monoclonal EL7 antibody (a gift from A. Pellicioli).

### FACS analysis

FACS analysis was carried out as previously described by staining cells with propidium iodide as described in [10], with the exception of the experiments presented in Figure 8, Figure S7, and Figure S12, where SYTOX green (Invitrogen) solution was used instead as described in [18].

### Extraction of replication intermediates and the 2D gel procedure

Purification of DNA intermediates and the 2D gel procedure were carried out as previously described [10], [17]. We note that each experiment was performed independently at least twice with qualitatively identical results and that a representative result is shown in the figures. The DNA samples were digested with *Hin*dIII and *Eco*RV and analyzed by 2D gel with probes against *ARS305* and/or the flanking region *ARS301*, or alternatively digested with *Nco*I and analyzed with probes recognizing *ARS305*.

### Quantification of replication intermediates

Quantification of signals of X-shaped intermediates was performed using the Image Quant software. For each time point, areas corresponding to the monomer spot (M), the X-spike signal and a region without any replication intermediates as background reference were selected and the signal intensities (SI) in percentage of each signal were obtained. The values for the X and monomer were corrected by subtracting from the SI value the background value after the latter was multiplied for the ratio between the dimension of the area for the intermediate of interest and for background. Thus, the values for X and M were calculated in the following way:
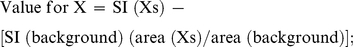


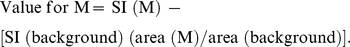



The relative signal intensity for the X was then determined by dividing the value for X with the sum of the total signals (the sum of the X and monomer values). The resulting values for X signals were then normalized and converted to percentage by using the highest value number of X for each experiment as 100 and normalizing the other values to it. At least three independent experiments conducted with isogenic strains were used for calculation of standard deviation. When mentioned, the value for the Y arc signal was calculated in a manner analogous to the one for the X, Value for Y  =  SI (Y) - [SI (background) (area (Y)/area (background))], and then the ratio X/Y was derived.

## Supporting Information

Figure S1 Formation of hemicatenane-like intermediates during damage-bypass processes. Replication forks encountering DNA damage can reprime downstream of the DNA lesion, leaving the DNA damage contained in a single stranded (ss) DNA gap behind the replication fork. This gap can be filled in using the newly synthesized DNA strand as a template, in a process referred to as template switch. We note that the term ‘template switch’ was used previously to describe other recombination processes involving a switch of templates, such as the homologous chromosome or other regions with microhomology. The hemicatenane-like intermediate generated in this process is visualized by 2D gel electrophoresis as an X-shaped intermediate. The resolution of these intermediates is mediated by Sgs1 and Top3. The accumulation of X-structures in sgs1 mutants requires Rad51 which could act either in promoting the formation of these intermediates via a process analogous with strand invasion or in the stabilization of the ssDNA regions contained in the X-structure in a paranemic junction or in a plectonemic junction if one of the strands is nicked.
(0.20 MB TIF)

Figure S2 The profiles of replication intermediates at *ARS305* from (A) wild type (FY1000) and *rad55Δ* (FY1066) and (B) wild type (FY1000), *rad55-S2*, *8*, *14A* (FY1068) and *rad59Δ* (FY1215) strains. The cells were synchronized in G2 prior to release in medium MMS 0.033% at 28°C.
(3.16 MB TIF)

Figure S3 (A) Wild type (W303-1A), *rfa1-t11* (HY1464), *sgs1* (HY1461) and *sgs1 rfa1-t11* (HY1459) were synchronized in α-factor and released in medium containing MMS 0.033% at 28°C. The replication intermediates were digested with *EcoRV* and *HindIII* and analyzed at the *ARS301* region. The quantification of the X-molecules and the ratio of X-molecules versus Y arcs, which represents the amount of X-molecules normalized to the ongoing replication in the analyzed genomic fragment, are shown.
(1.47 MB TIF)

Figure S4 2D gel analysis at *ARS305* region of replication intermediates digested with *NcoI* from wild type (W303-1A) and exo1Δ (HY1463). The cells were synchronized with nocodazole and released in medium containing MMS 0.033% at 28°C.
(0.68 MB TIF)

Figure S5 Damage sensitivity of polymerase mutants. Spot assays of strains (A) wild type (FY0100), *cdc2-1* (FY0107), *pol32Δ* (FY0106), *pol3-ct* (FY1174), (B) wild type (FY1274), *pol2-11* (FY1275) and (C) wt (FY1000), *rev7Δ rev1Δ rad30Δ* (HY1466) at different concentrations of MMS at 28°C.
(1.32 MB TIF)

Figure S6 The replication intermediates from wild type (FY1000), *rad30Δ* (CY7715), and *rev7Δ rev1Δ rad30Δ* (HY1466) cells were digested with *EcoRV* and *HindIII* and analyzed at the *ARS305* region.
(2.29 MB TIF)

Figure S7 (A) Viability of wild type (FY0100), *cdc2-1* (FY0107), *sgs1Δ* (HY0100), *cdc2-1 sgs1Δ* (FY0107) at 25°C, 30°C, and 37°C as measured by spot assay. (B) FACS profile of the same strains as in (A) grown at 25°C to log phase then shifted for 3 hours to either 25°C or 30°C.
(1.20 MB TIF)

Figure S8 The replication intermediates from (A) wild-type (FY1274), *pol2-11* (FY1275), and (B) wild type (FY0100), *cdc2-1* (FY0107) cells were digested with *EcoRV* and *HindIII* and analyzed at the *ARS305* region.
(1.09 MB TIF)

Figure S9 The effect of the *cdc2-1* mutation on the X versus Y arc structures. The experiments were performed at the semi-permissive temperature of 30°C as described in Figure 6B. The X and Y arc values were calculated as described in the Experimental Procedures. The X/Y ratio, the ratio of X-molecules versus Y arcs, which represents the amount of X-molecules normalized to the ongoing replication in the analyzed genomic fragment, at different time points with standard deviations is plotted for *sgs1 CDC2+* and *sgs1 cdc2-1* cells.
(0.15 MB TIF)

Figure S10 2D gel analysis of replication intermediates from wild-type (FY0100), *cdc2-1* (FY0107), *sgs1* (HY0100) and *sgs1 cdc2-1* (HY0103) cells. The experiments were performed at the semi-permissive temperature of 30°C. The DNA samples were digested with *HindIII* and *EcoRV* and the membranes hybridized with a probe corresponding to *ARS301*. Quantification of X-molecules and the X/Y ratio, which represents the amount of X-molecules normalized to the ongoing replication in the analyzed genomic fragment, at different time points is shown.
(1.51 MB TIF)

Figure S11 2D gels analysis of replication intermediates forming at *ARS305* from wild type (FY1000) and *pol3-ct* (FY1174) cells replicating in the presence of MMS damage at the permissive temperature of 30°C. The replication intermediates were digested with *HindIII* and *EcoRV*.
(1.24 MB TIF)

Figure S12 2D gel analysis of replication intermediates from wild-type (FY0108) and *pol32Δ* (FY1379). The cells were grown in YP media containing 0.2% galactose and 1.8% raffinose at 30°C, arrested with α-factor, then released in YP media containing 0.5% galactose and 1.5% raffinose and MMS 0.033% at 25°C. The replication intermediates were digested with *HindIII* and *EcoRV*, and analyzed with a probe corresponding to *ARS305*.
(2.84 MB TIF)

Figure S13 Exo1 does not affect MMS-induced Rad53 activation Exponentially growing wild type (W303-1A) and *exo1* (HY1463) cells were treated for 2 and 4 hours with MMS at two different concentrations, 0.01% and 0.02%. Western blot analysis was performed to detect Rad53 phosphorylation.
(0.24 MB TIF)

Table S1 List of strains used in this study.
(0.07 MB DOC)
