# Supplementary figures and images for: Replication and Recombination Factors Contributing to Recombination-Dependent Bypass of DNA Lesions by Template Switch
Source: PLoS Genet. 2010 Nov 11;6(11):e1001205. doi: 10.1371/journal.pgen.1001205 (PMC2978687; doi:10.1371/journal.pgen.1001205)

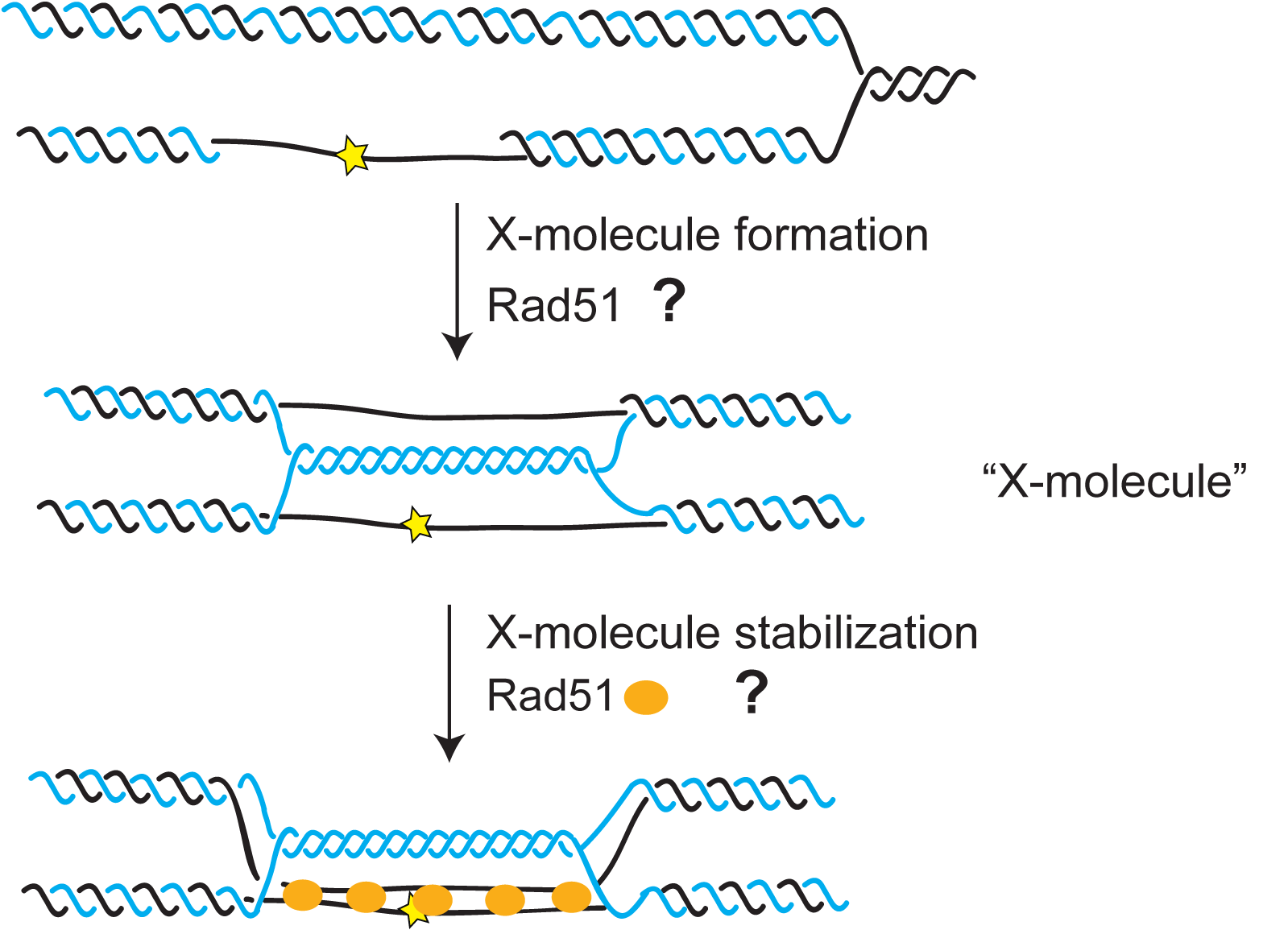

Supplement: Figure S1 — Formation of hemicatenane-like intermediates during damage-bypass processes. Replication forks encountering DNA damage can reprime downstream of the DNA lesion, leaving the DNA damage contained in a single stranded (ss) DNA gap behind the replication fork. This gap can be filled in using the newly synthesized DNA strand as a template, in a process referred to as template switch. We note that the term ‘template switch’ was used previously to describe other recombination processes involving a switch of templates, such as the homologous chromosome or other regions with microhomology. The hemicatenane-like intermediate generated in this process is visualized by 2D gel electrophoresis as an X-shaped intermediate. The resolution of these intermediates is mediated by Sgs1 and Top3. The accumulation of X-structures in sgs1 mutants requires Rad51 which could act either in promoting the formation of these intermediates via a process analogous with strand invasion or in the stabilization of the ssDNA regions contained in the X-structure in a paranemic junction or in a plectonemic junction if one of the strands is nicked. (0.20 MB TIF) [file pgen.1001205.s001.tif]

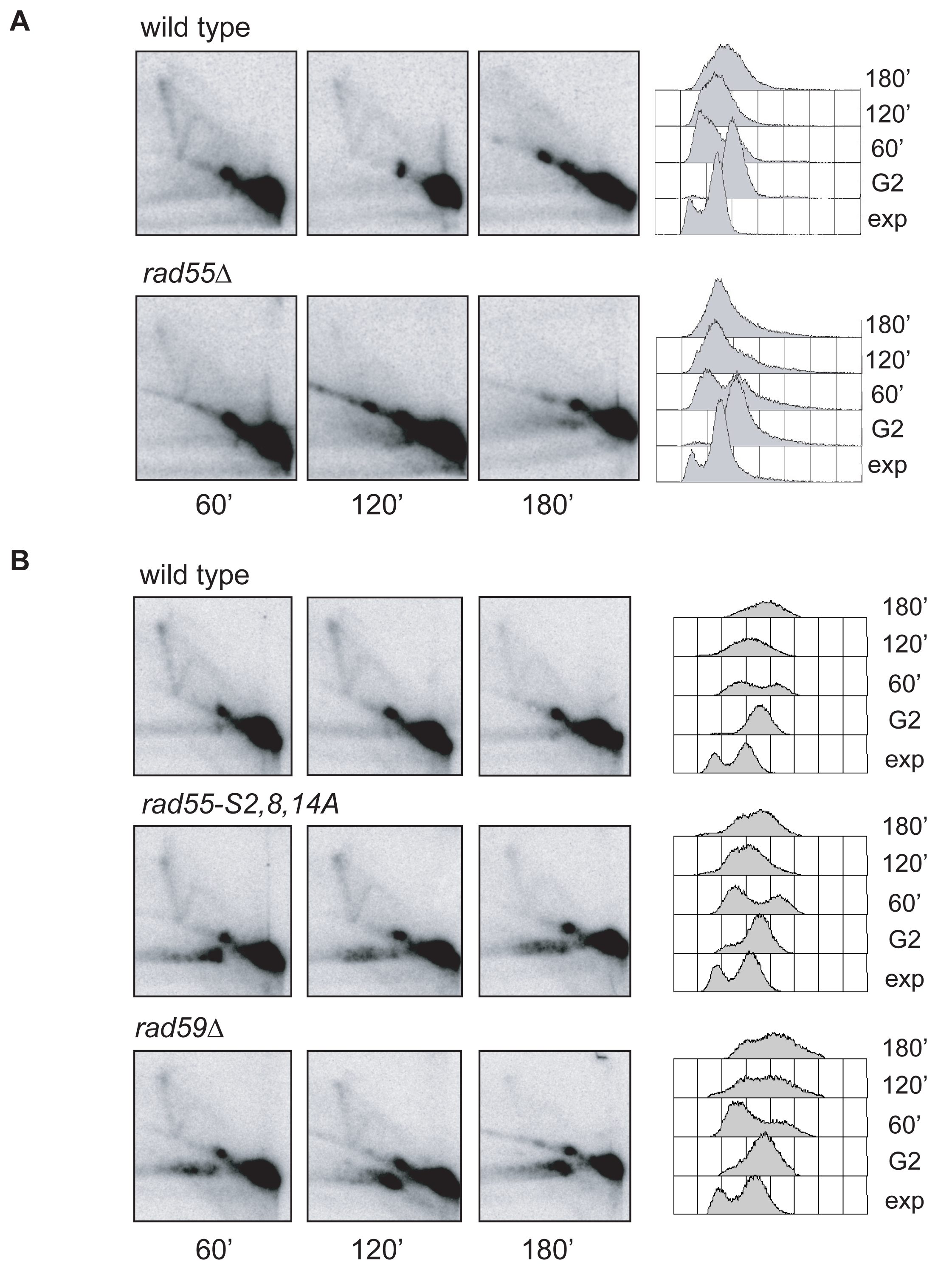

Supplement: Figure S2 — The profiles of replication intermediates at ARS305 from (A) wild type (FY1000) and rad55Δ (FY1066) and (B) wild type (FY1000), rad55-S2, 8, 14A (FY1068) and rad59Δ (FY1215) strains. The cells were synchronized in G2 prior to release in medium MMS 0.033% at 28°C. (3.16 MB TIF) [file pgen.1001205.s002.tif]

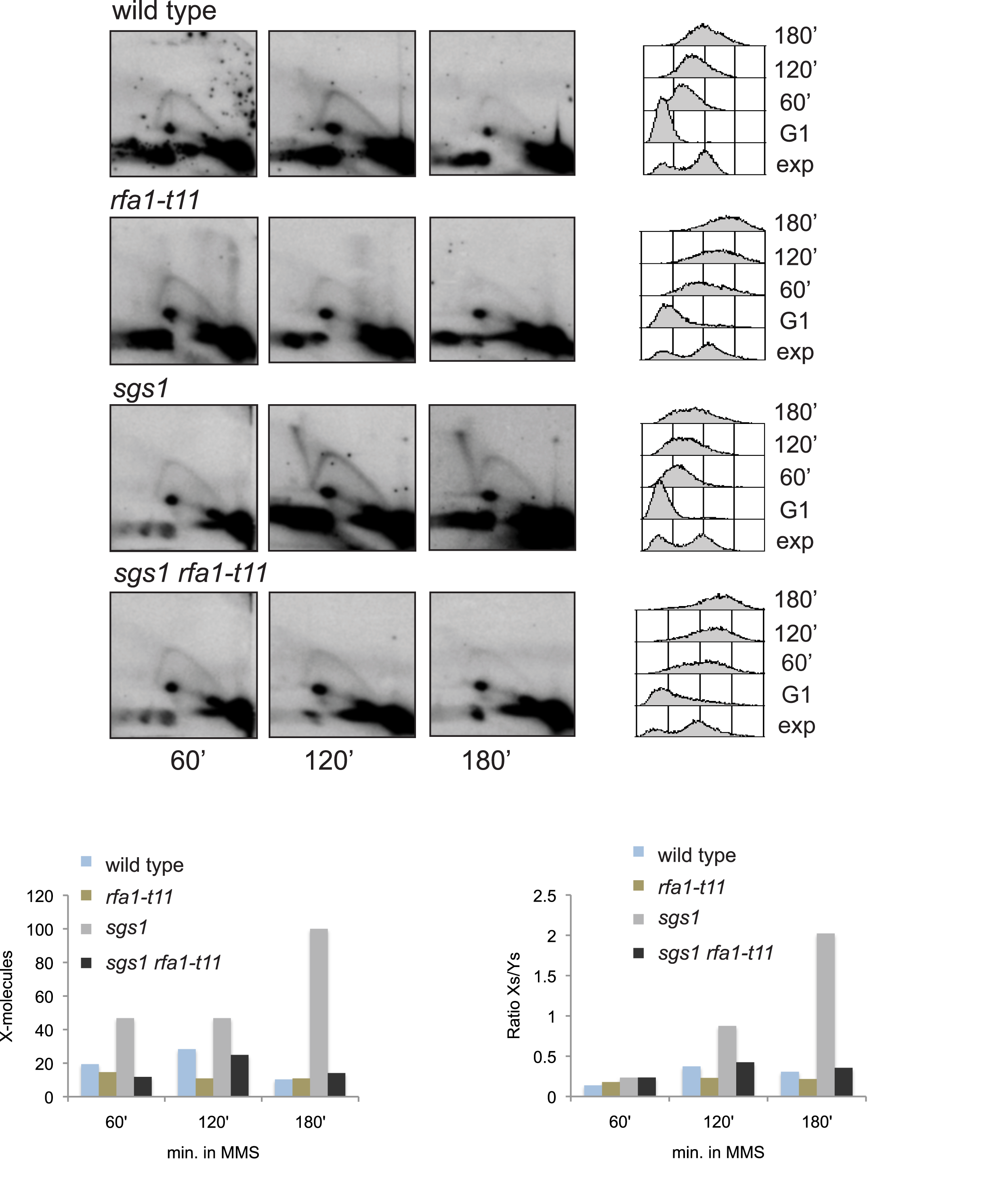

Supplement: Figure S3 — (A) Wild type (W303-1A), rfa1-t11 (HY1464), sgs1 (HY1461) and sgs1 rfa1-t11 (HY1459) were synchronized in α-factor and released in medium containing MMS 0.033% at 28°C. The replication intermediates were digested with EcoRV and HindIII and analyzed at the ARS301 region. The quantification of the X-molecules and the ratio of X-molecules versus Y arcs, which represents the amount of X-molecules normalized to the ongoing replication in the analyzed genomic fragment, are shown. (1.47 MB TIF) [file pgen.1001205.s003.tif]

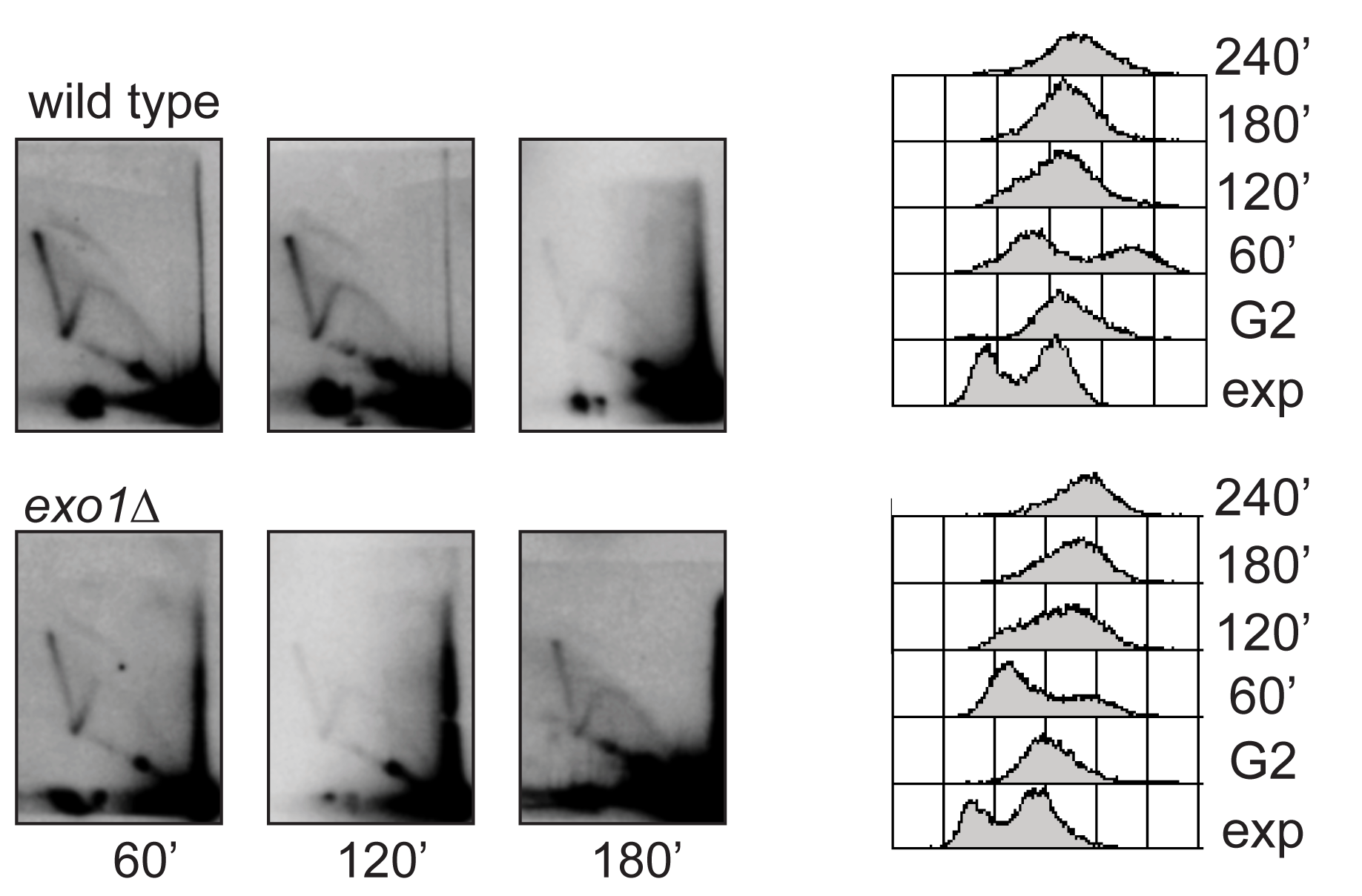

Supplement: Figure S4 — 2D gel analysis at ARS305 region of replication intermediates digested with NcoI from wild type (W303-1A) and exo1Δ (HY1463). The cells were synchronized with nocodazole and released in medium containing MMS 0.033% at 28°C. (0.68 MB TIF) [file pgen.1001205.s004.tif]

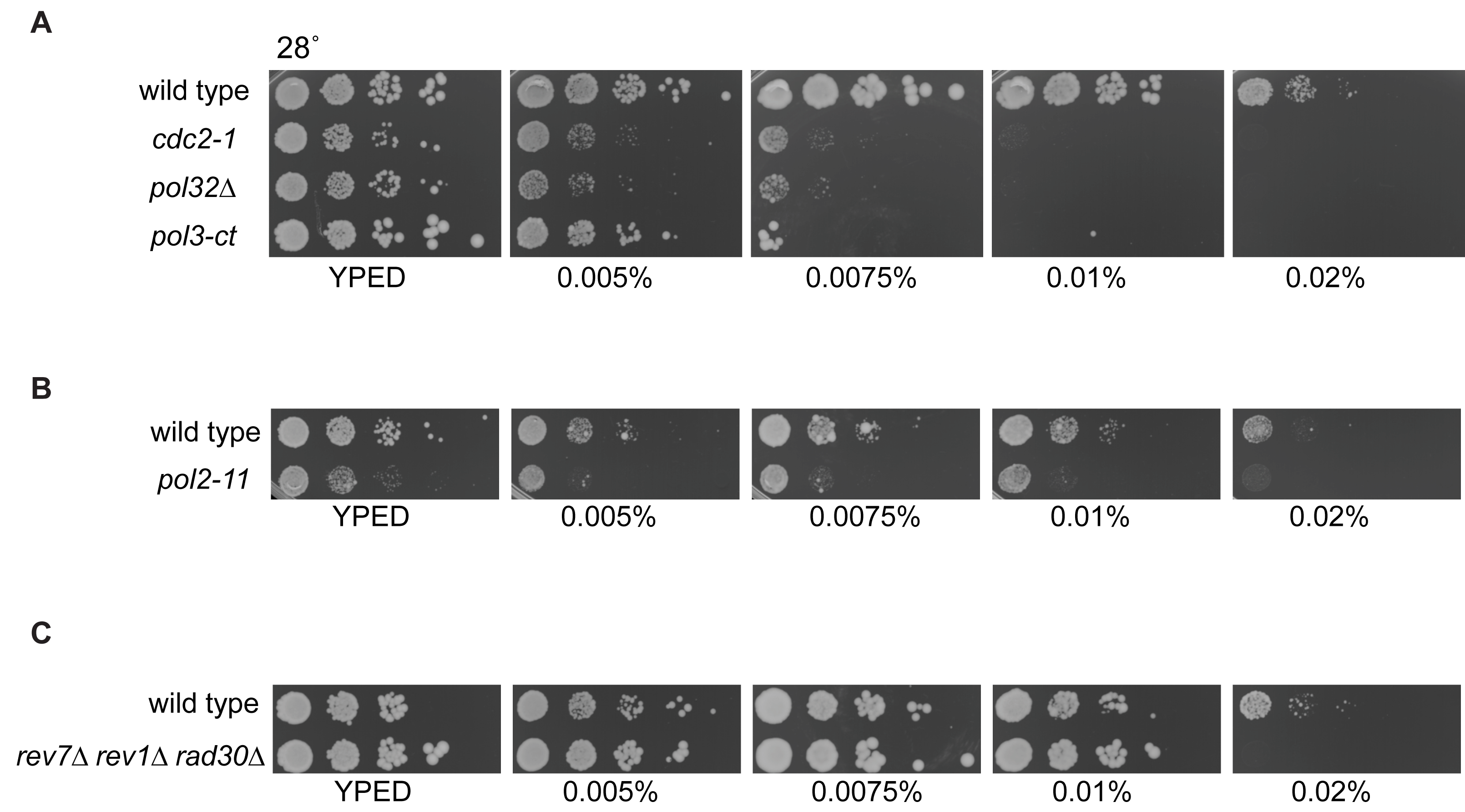

Supplement: Figure S5 — Damage sensitivity of polymerase mutants. Spot assays of strains (A) wild type (FY0100), cdc2-1 (FY0107), pol32Δ (FY0106), pol3-ct (FY1174), (B) wild type (FY1274), pol2-11 (FY1275) and (C) wt (FY1000), rev7Δ rev1Δ rad30Δ (HY1466) at different concentrations of MMS at 28°C. (1.32 MB TIF) [file pgen.1001205.s005.tif]

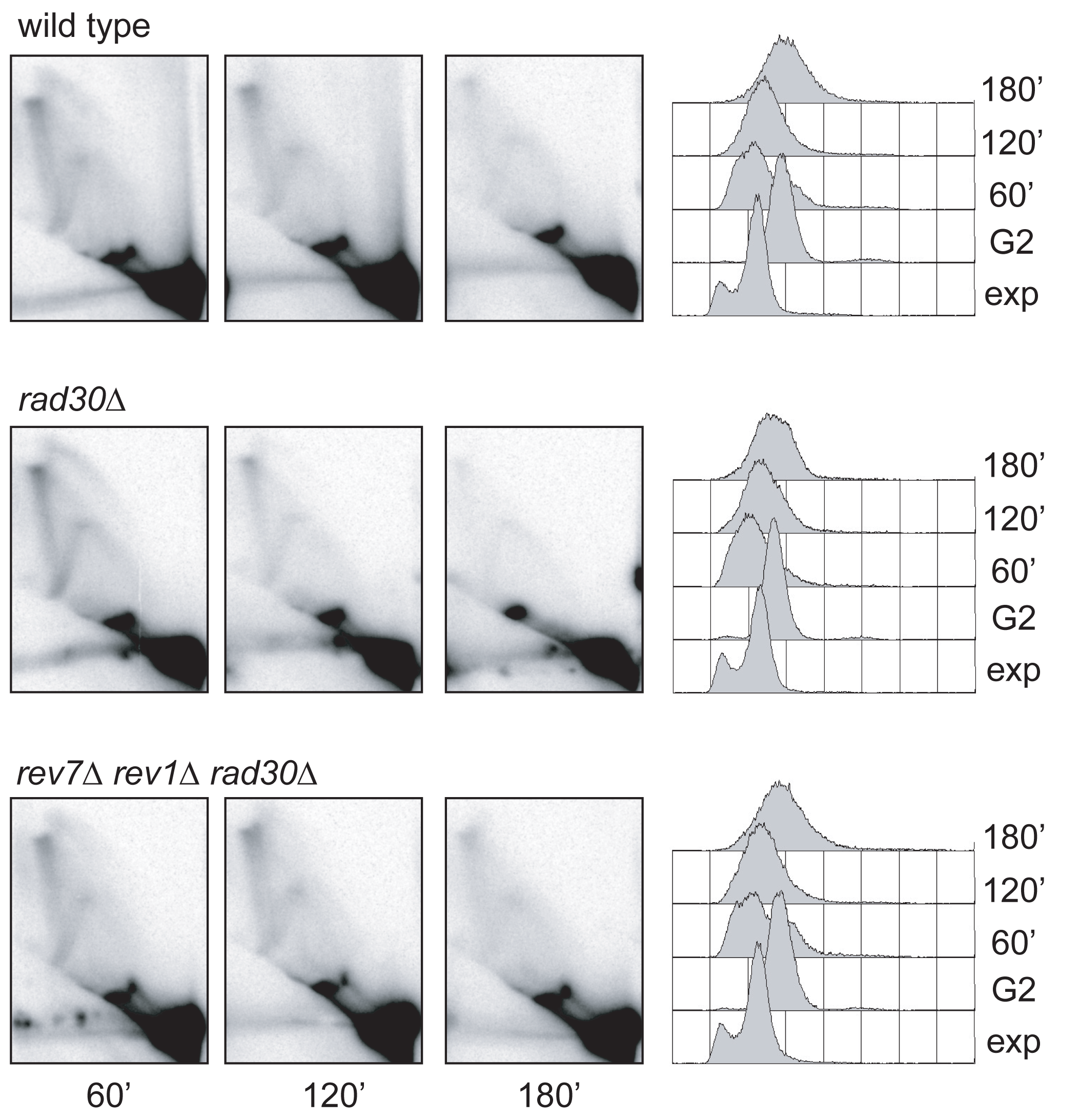

Supplement: Figure S6 — The replication intermediates from wild type (FY1000), rad30Δ (CY7715), and rev7Δ rev1Δ rad30Δ (HY1466) cells were digested with EcoRV and HindIII and analyzed at the ARS305 region. (2.29 MB TIF) [file pgen.1001205.s006.tif]

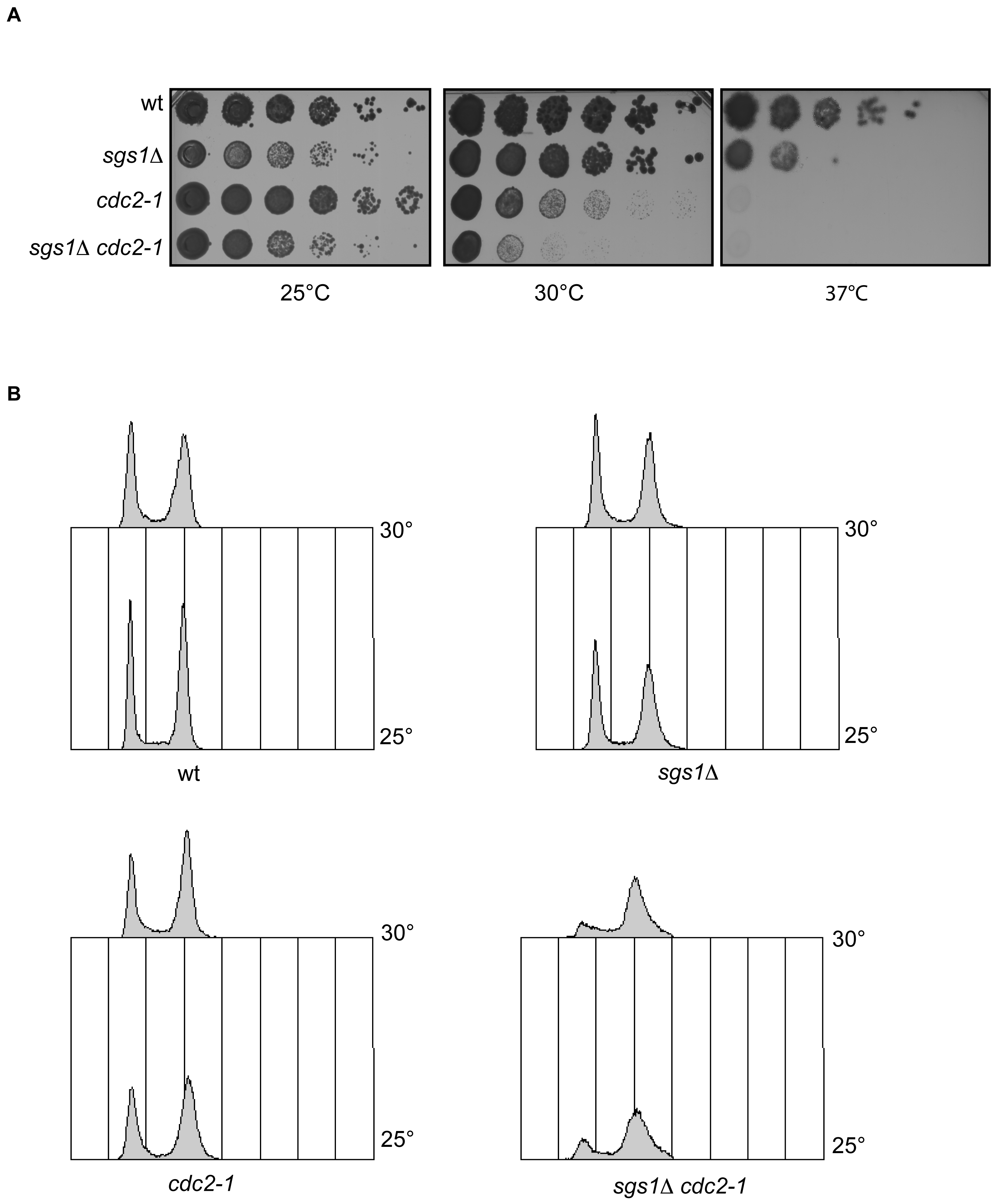

Supplement: Figure S7 — (A) Viability of wild type (FY0100), cdc2-1 (FY0107), sgs1Δ (HY0100), cdc2-1 sgs1Δ (FY0107) at 25°C, 30°C, and 37°C as measured by spot assay. (B) FACS profile of the same strains as in (A) grown at 25°C to log phase then shifted for 3 hours to either 25°C or 30°C. (1.20 MB TIF) [file pgen.1001205.s007.tif]

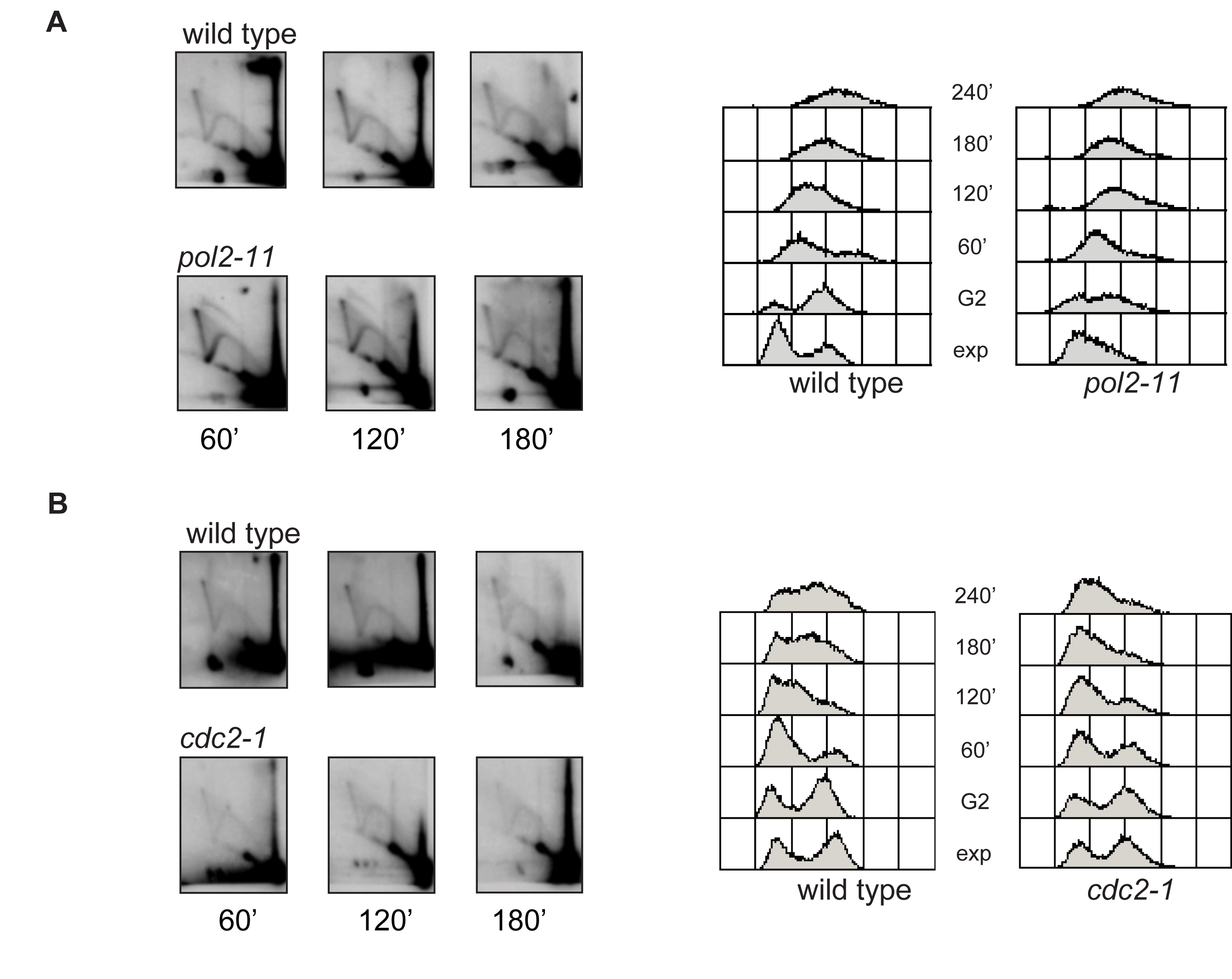

Supplement: Figure S8 — The replication intermediates from (A) wild-type (FY1274), pol2-11 (FY1275), and (B) wild type (FY0100), cdc2-1 (FY0107) cells were digested with EcoRV and HindIII and analyzed at the ARS305 region. (1.09 MB TIF) [file pgen.1001205.s008.tif]

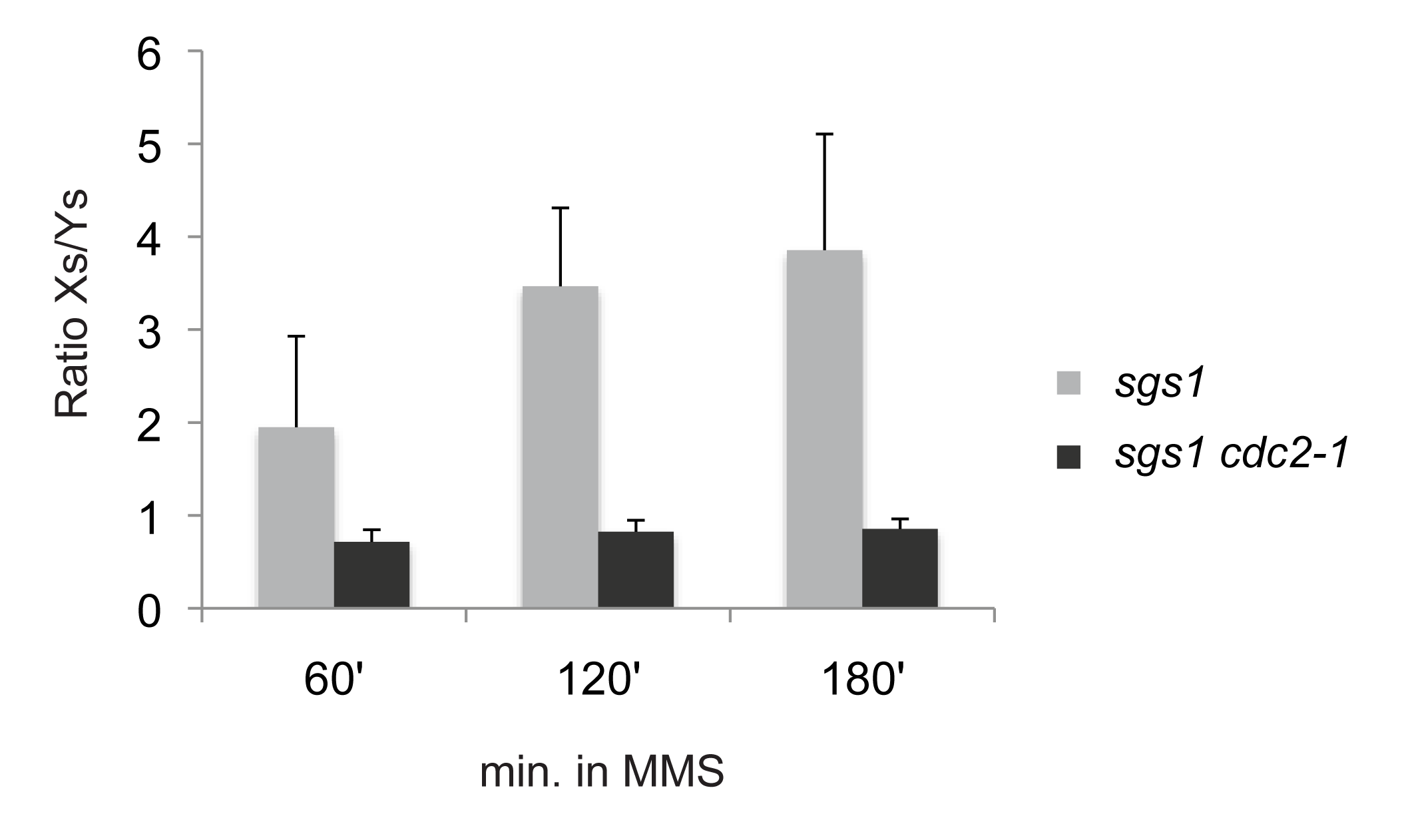

Supplement: Figure S9 — The effect of the cdc2-1 mutation on the X versus Y arc structures. The experiments were performed at the semi-permissive temperature of 30°C as described in Figure 6B. The X and Y arc values were calculated as described in the Experimental Procedures. The X/Y ratio, the ratio of X-molecules versus Y arcs, which represents the amount of X-molecules normalized to the ongoing replication in the analyzed genomic fragment, at different time points with standard deviations is plotted for sgs1 CDC2+ and sgs1 cdc2-1 cells. (0.15 MB TIF) [file pgen.1001205.s009.tif]

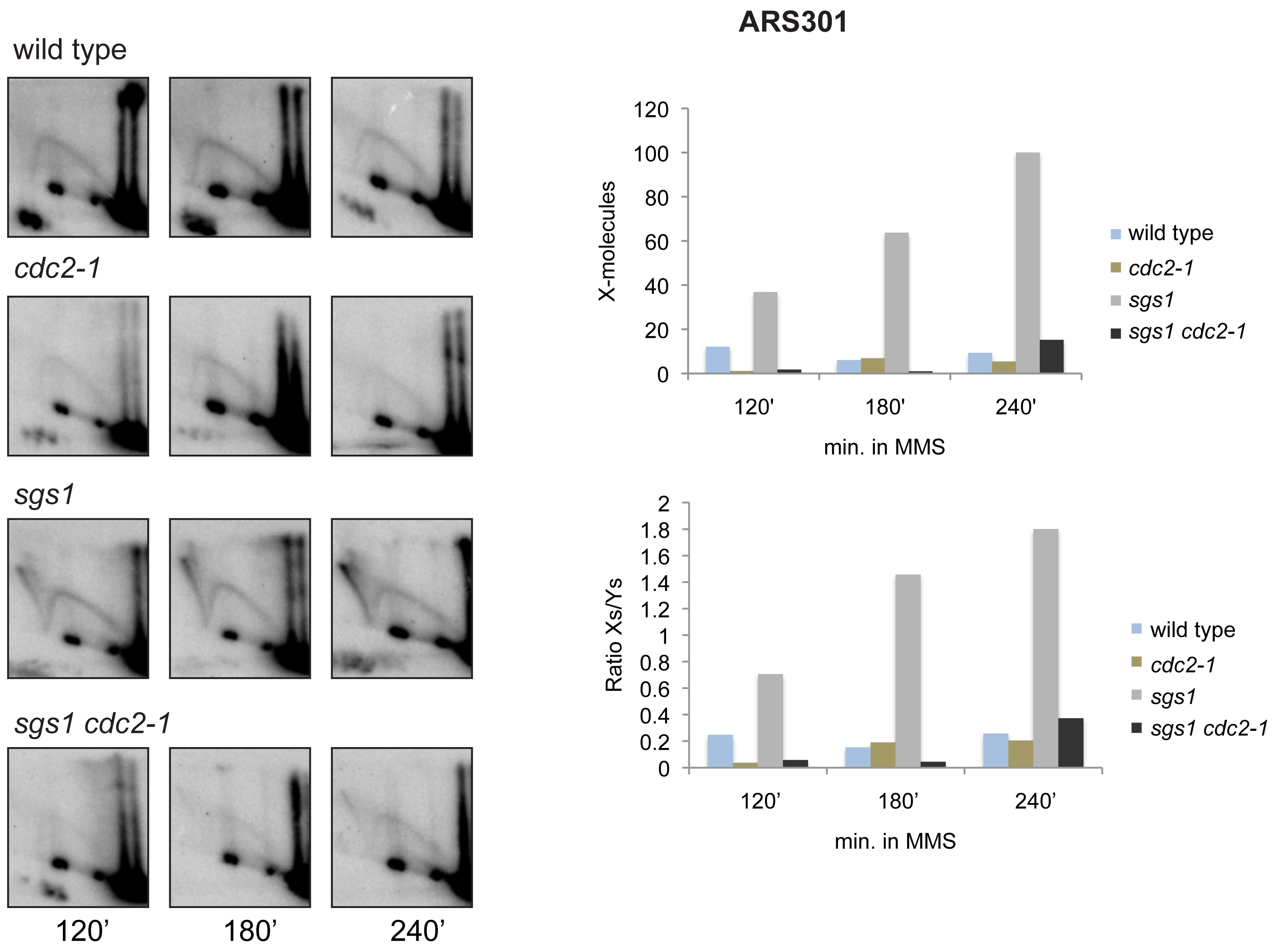

Supplement: Figure S10 — 2D gel analysis of replication intermediates from wild-type (FY0100), cdc2-1 (FY0107), sgs1 (HY0100) and sgs1 cdc2-1 (HY0103) cells. The experiments were performed at the semi-permissive temperature of 30°C. The DNA samples were digested with HindIII and EcoRV and the membranes hybridized with a probe corresponding to ARS301. Quantification of X-molecules and the X/Y ratio, which represents the amount of X-molecules normalized to the ongoing replication in the analyzed genomic fragment, at different time points is shown. (1.51 MB TIF) [file pgen.1001205.s010.tif]

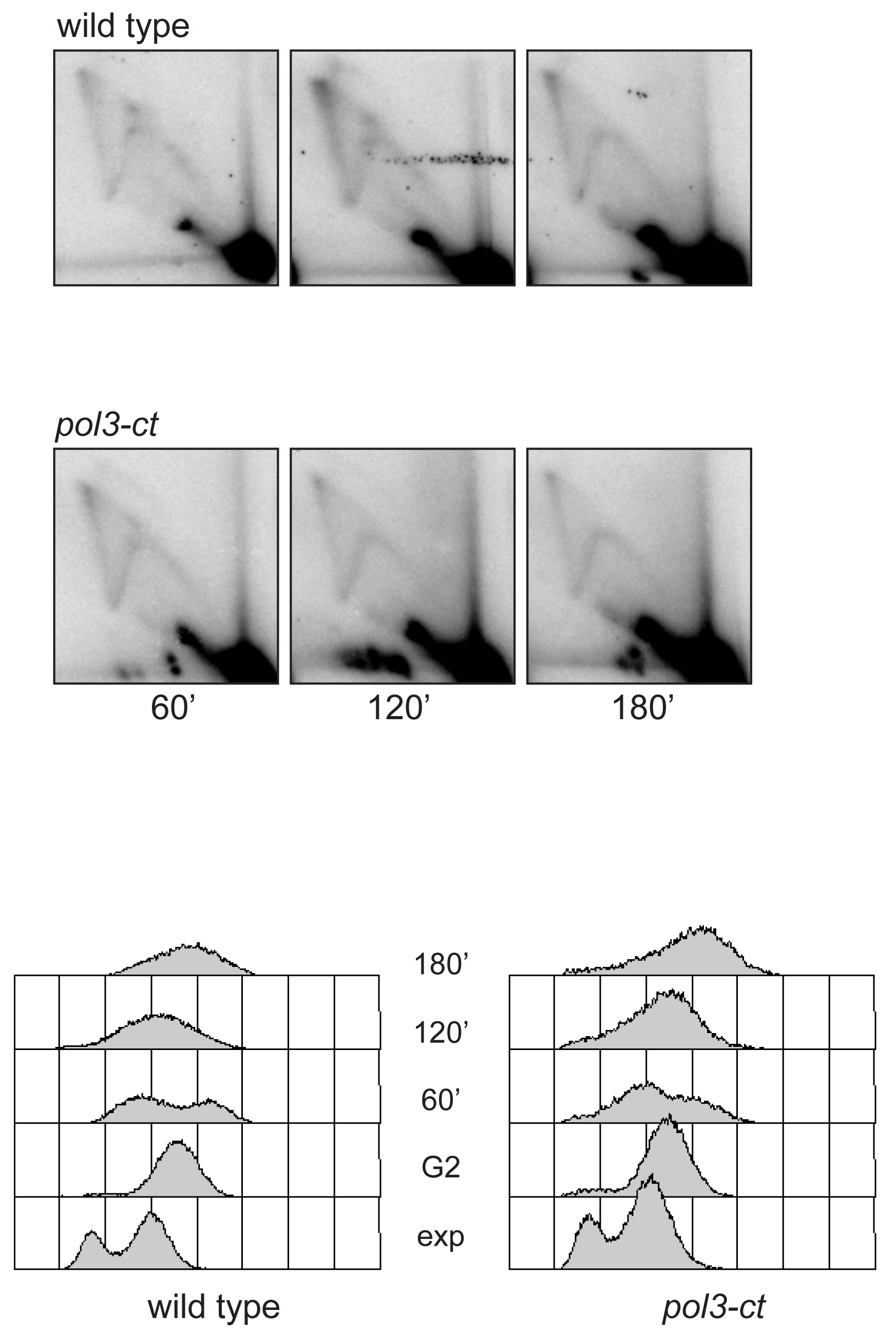

Supplement: Figure S11 — 2D gels analysis of replication intermediates forming at ARS305 from wild type (FY1000) and pol3-ct (FY1174) cells replicating in the presence of MMS damage at the permissive temperature of 30°C. The replication intermediates were digested with HindIII and EcoRV. (1.24 MB TIF) [file pgen.1001205.s011.tif]

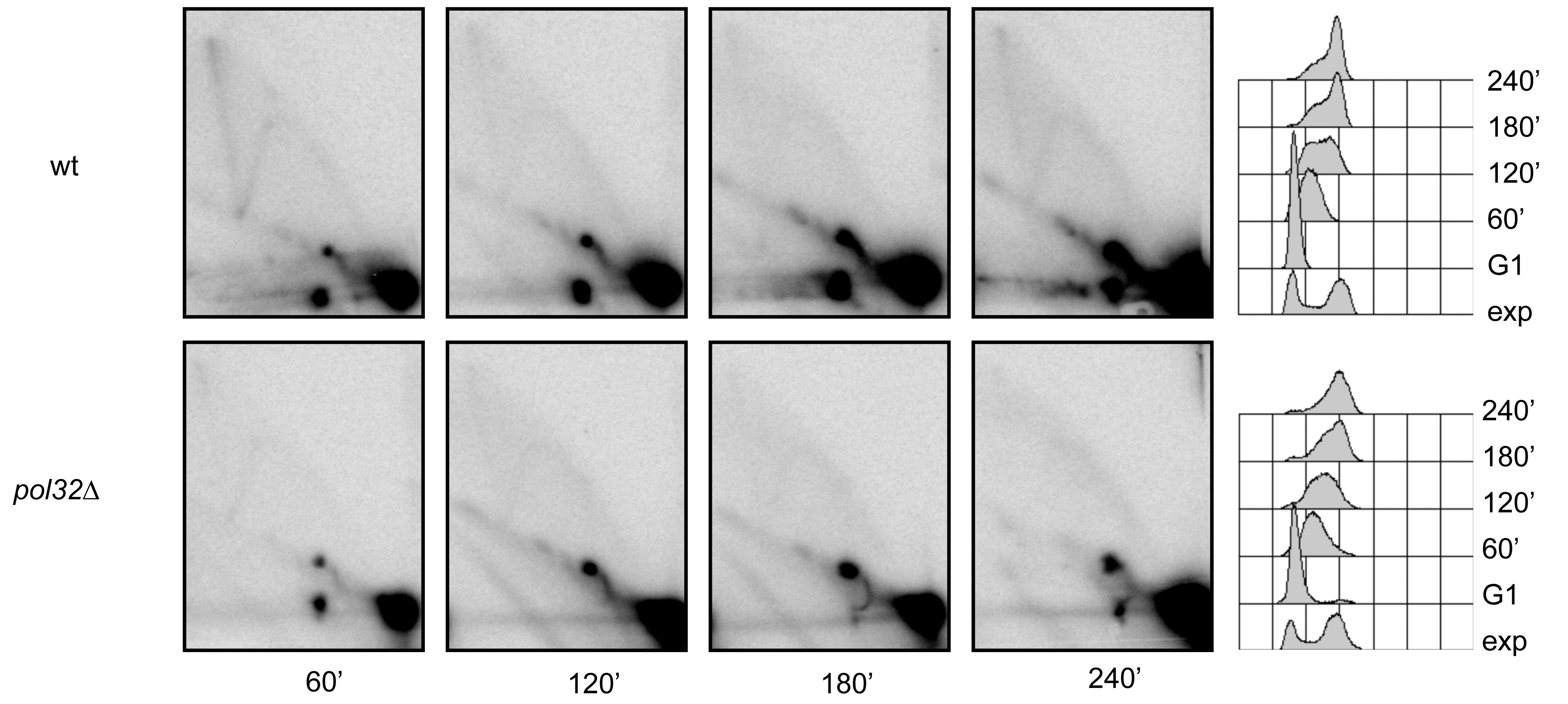

Supplement: Figure S12 — 2D gel analysis of replication intermediates from wild-type (FY0108) and pol32Δ (FY1379). The cells were grown in YP media containing 0.2% galactose and 1.8% raffinose at 30°C, arrested with α-factor, then released in YP media containing 0.5% galactose and 1.5% raffinose and MMS 0.033% at 25°C. The replication intermediates were digested with HindIII and EcoRV, and analyzed with a probe corresponding to ARS305. (2.84 MB TIF) [file pgen.1001205.s012.tif]

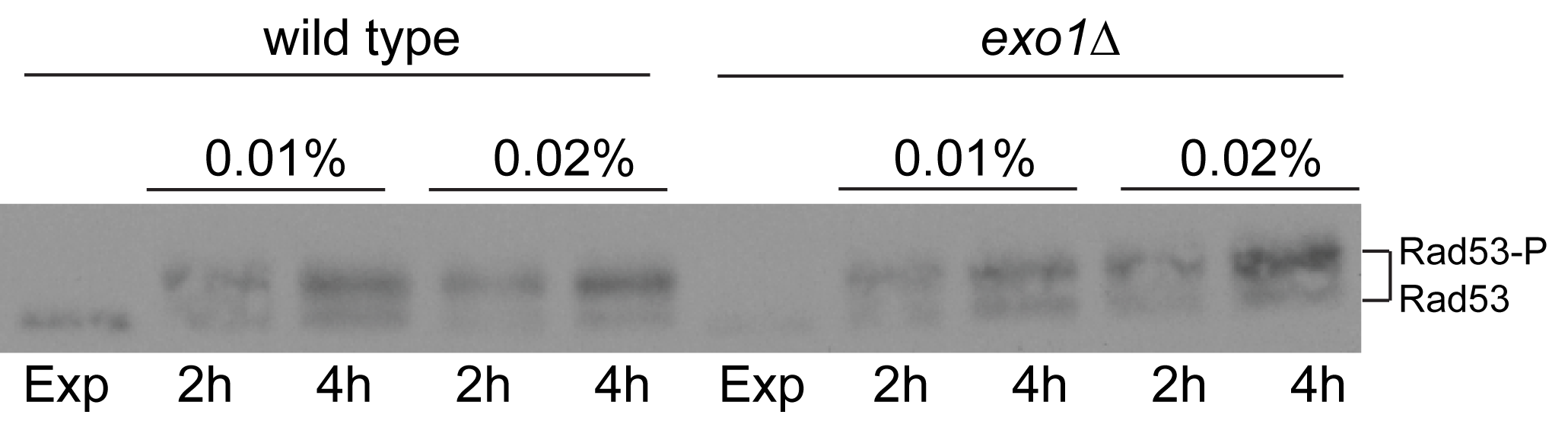

Supplement: Figure S13 — Exo1 does not affect MMS-induced Rad53 activation Exponentially growing wild type (W303-1A) and exo1 (HY1463) cells were treated for 2 and 4 hours with MMS at two different concentrations, 0.01% and 0.02%. Western blot analysis was performed to detect Rad53 phosphorylation. (0.24 MB TIF) [file pgen.1001205.s013.tif]
