# Supplementary material for: Replication and Recombination Factors Contributing to Recombination-Dependent Bypass of DNA Lesions by Template Switch
Source: PLoS Genet. 2010 Nov 11;6(11):e1001205. doi: 10.1371/journal.pgen.1001205 (PMC2978687; doi:10.1371/journal.pgen.1001205)
Supplement: Table S1 — List of strains used in this study. (0.07 MB DOC) [file pgen.1001205.s014.doc]

## F. Vanoli *et. al*

Table S1. List of Strains Used in This Study

| W303-1A  CY7069 | *MAT***a***, ade2-1, ura3, trp1-1, leu2-3,112, his3-11,15, can1-100, GAL, rad5-535* | R.Rothstein |
| --- | --- | --- |
| FY1000  (W303) | *MAT***a***, ade2-1, ura3, trp1-1, leu2-3,112, his3-11,15, can1-100, GAL, PSI+, RAD5+* | Lab. Stock |
| HY1461 | W303-1A *sgs1::AUR1-C* | This Study |
| HY1463 | W303-1A *exo1Δ::KANMX6* | Lab. Stock |
| HY1448 | W303-1A *sgs1::AUR1-C exo1Δ::KanMX4* | This Study |
| HY1464 | W303-1A *rfa1-t11* | Lab. Stock |
| HY1459 | W303-1A *sgs1::AUR1-C rfa1-t11* | This Study |
| FY1058 | W303-1A *sgs1Δ::KanMX4* | Lab. Stock |
| HY1460 | FY1000 *sgs1Δ::NATMX4 rad55Δ::KURA3* | This Study |
| HY0799 | FY1000 *sgs1Δ:: KanMX4 rad55-S2,8,14A* | This Study |
| FY1066 | FY1000 *rad55Δ::KURA3* | W. Heyer |
| FY1068 | FY1000 *rad55-S2,8,14A* | W. Heyer |
| FY1215 | FY1000 *rad59Δ::LEU2* | Lab. Stock |
| HY1414 | *MAT***a**, *ade2-1, trp1-1, leu2-3112, his3-1115, ura3, can1-100 GAL PSI1+ rad59Δ::LEU2 sgs1Δ::NATMX4* | This Study |
| HY1465 | FY1000 *sgs1Δ::HIS3* | This Study |
| HY1467 | FY1000 *sgs1Δ::HIS3 rad30Δ::KanMX6* | This Study |
| HY1466 | FY1000 *rev7:: KanMX4 rev1:: TRP1 rad30Δ::TRP1* | This Study |
| CY7715 | FY1000 *rad30Δ::KanMX6* | This Study |
| HY1466 | FY1000 *rev7:: KanMX4 rev1:: TRP1 rad30Δ::TRP1* | This Study |
| HY1468 | FY1000 *sgs1Δ::HIS3* *rev7:: KanMX4 rev1:: TRP1 rad30Δ::TRP1* | This Study |
| FY0100 | *MAT***alpha***, ade2-101, ura3-52, leu2-3,112, lys2-801 his3-∆* | P. Burgers |
| FY0107 | *MAT***alpha** *his3-Δ2002-101 leu2-3, 112 ura3-52 trp1Δ can1 cdc2-1 pol32Δ::HIS3 (pBL389[POL32-URA3])* | P. Burgers |
| HY0100 | FY0100 *sgs1Δ::AUR1-C* | This Study |
| HY0103 | FY0107 *sgs1Δ::AUR1-C* | This Study |
| FY1274 | *MAT***a** *trp-1-289, ura3-12, ade2-101 GAL2 can1* | H. Araki |
| FY1275 | FY1274 *pol2-11* | H. Araki |
| HY1455 | FY1274 *sgs1::AUR1-C* | This Study |
| HY1456 | FY1274 *sgs1::AUR1-C pol2-11* | This Study |
| FY1174 | W303 (*MAT***a**, *ade2-1, TRP1^+^ leu2-3,112, his3-11,15, ura3,can1-100, LYS2^+^)* *pol3-ct* | L. Maloisel |
| HY1257 | FY1174 *sgs1::AUR1-C* | L. Maloisel |
| FY0106 | *MAT***a** *ura3-52 his3-delta200 leu2-3,112 lys2-801 trp1 tyr1 can1 pol32 Δ:HIS3* | P. Burgers |
| FY0108 | *MAT***a** *his3-delta200 leu2-3, 112 lys-801 trp1-1 (am) ura3-52* | S. Jentsch |
| FY1359 | FY0108  *pGAL-3HA-SGS1::natNT2* | S.Jentsch |
| FY1379 | FY0108 *pol32::klTRP1 pGAL-HA3SGS1::natNT2* | S.Jentsch |
